# Reducing Food Loss and Associated Greenhouse Gas Emissions Using a Dynamic Shelf Life Approach

**DOI:** 10.1021/acs.est.5c04093

**Published:** 2025-06-30

**Authors:** Junzhang Wu, Yifeng Zou, Gang Liu, Li Xue, Zhimin Shi, Andrea Fedele, Alessandro Manzardo

**Affiliations:** † CESQA (Quality and Environmental Research Center), Department of Civil, Environmental and Architectural Engineering, University of Padova, Via Marzolo 9, 35131 Padova, Italy; ‡ School of Management, Guangzhou University, Guangzhou 510006, China; § College of Urban and Environmental Sciences, 12465 Peking University, Beijing 100871, China; ∥ College of Economics and Management, 34752 China Agricultural University, Beijing 100083, China; ¶ Academy of Global Food Economics and Policy, 34752 China Agricultural University, Beijing 100083, China; # State Key Joint Laboratory of Environmental Simulation and Pollution Control, School of Environment, 47836 Beijing Normal University, Beijing 100875, China

**Keywords:** expiry date, food loss and waste, greenhouse gas (GHG), Internet of Things (IoT), sensor, life cycle assessment (LCA)

## Abstract

The integration of IoT sensors with dynamic shelf life (DSL) systems unlocks real-time visibility into perishable goods, yet the full life-cycle trade-offs of such technologies remain underexplored. This study develops a process-based life cycle framework, incorporating a kinetic quality-degradation model and Monte Carlo simulations, to evaluate both avoided food loss and waste and sensor-embedded climate burdens across China’s fresh food chains. Results show this IoT-DSL regime extends average shelf life by 8.1–13.8% in fruits, dairy, and vegetables, although gains fall two- to 5-fold for animal and aquatic products at lower quality thresholds, while nontechnical interventions deliver only 3.2–6.5% waste reductions. Large-scale IoT-DSL deployment could avert 17.32 ± 3.65 Mt yr^–1^ of waste and achieve a net cut of 51.00 ± 10.38 Mt CO_2_-eq yr^–1^ (≈10.9% of China’s food-chain emissions), despite introducing 7.7 Mt CO_2_-eq yr^–1^ from sensors. Upstream, sensor fabrication dominates impacts, underscoring the need for eco-designed materials and robust e-waste recovery. Sensitivity analysis identifies production emission intensity, inherent shelf life, and logistics crate capacity as critical drivers. Projected improvements in the input–output efficiency indicator—from 17.5 in 2020 to 18.9 by 2030—and future scenarios incorporating food-tech innovations and plant-based dietary shifts underscore further mitigation potential.

## Graphical abstract

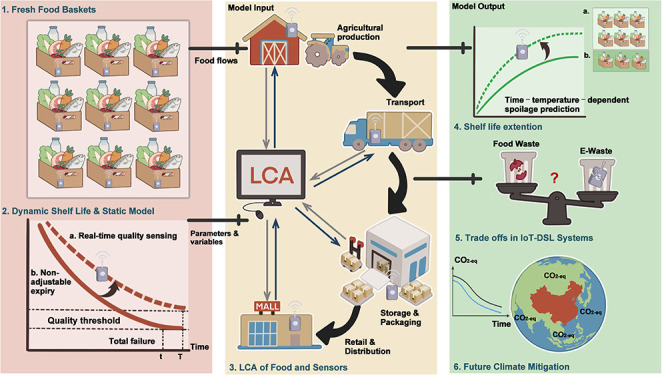

## 1 Introduction

Food loss and waste (FLW) are responsible for significant to global greenhouse gas (GHG) emissions, contributing approximately 3.3 Gt CO_2_-eq annually—equivalent to 6% of global emissions.^1^ When accounting for land use changes and deforestation, this impact exceeds 4.4 Gt CO_2_-eq, highlighting the critical need to address FLW as part of global climate mitigation strategies.^2^ Technological advancements in food supply chains present an opportunity to address this challenge by extending the shelf life of perishable goods. A study by the European Union identified date labeling inaccuracies as a significant driver of food waste, responsible for approximately 10% (∼8.9 Mt/year) of the total FLW across EU-28 countries (∼88 Mt/year).^3^ Considering product shelf life has a great influence on FLW reduction, a sensor-based dynamic shelf life (DSL) approach has been targeted as a key means for efficient waste-prevention measures,^3^ thus providing savings in embodied GHG emissions.

The advancement of emerging information technologies, including the Internet of Things (IoT),^4^ radio frequency identification,^5^ wireless sensor network,^6^ machine learning,^7^ big data,^8^ cloud storage,^9^ and digital twins,^10^ has revolutionized efforts to reduce FLW by extending shelf life of perishable foods across temperature-sensitive supply chains. Shelf life, defined as the period during which food remains acceptable for consumption, may now be dynamically managed through IoT sensor-enabled solutions.^11^ This IoT sensor-enabled sensors track food trajectories from farm to fork and monitor critical parameters—such as temperature, humidity, gas emissions, and motion—during transit and storage.^4,5,12^ By leveraging quality-decay models that correlate shelf life with temperature and time, these technologies enable real-time adjustments to expiration dates based on dynamic thermal conditions and quality loss throughout temperature-sensitive supply chains.^13^ Addressing the asymmetry of shelf life information in this manner facilitates accurate predictions of remaining shelf life, significantly reducing FLW.^14^ As a result, this innovative IoT-DSL approach provides food stakeholders with transparent, data-driven insights, surpassing the limitations of conventional static shelf life determinations.^3,15^ It promotes interconnectivity among suppliers, logistics, and retailers while enabling evidence-based policymaking. Supporting Information (SI) Section 1 provides a comprehensive review of IoT sensor impacts on FLW reduction from an environmental perspective.

Despite advancements, two critical research gaps persist in evaluating IoT sensor-based DSL systems. First, large-scale assessments often overestimate the emissions mitigation potential of IoT systems by attributing all FLW reductions to IoT sensor technologies without accounting for the critical role of cold chain infrastructure, such as refrigerated storage and transport. This oversight inflates the perceived impact of IoT systems, particularly in regions with underdeveloped cold chains.^4^ Second, environmental assessments frequently conflate food loss and food waste impacts, applying uniform models that overlook the dynamic nature of food quality deterioration. Food loss, involving gradual quality decline, requires distinct modeling compared to food waste, which reflects the complete disposal of items and their full carbon footprint. Here, our proposed sensor-enabled IoT-DSL system primarily addresses food waste at the retail stage, which has a greater environmental burden than food loss earlier in the supply chain. Hence, empirical models further complicate evaluations by oversimplifying the drivers of retail food waste, such as temperature variability, initial food quality, and consumer behavior. This can lead to both overestimations, by ignoring cold chain contributions, and underestimations, by failing to capture the complex interdependencies influencing waste reduction.

This analysis develops a computer-simulated, multicommodity accounting framework to quantify avoidable FLW from “farm-to-retail” by embedding a sensor-based DSL system within China’s fresh food supply chains. The central focus of this study is determining whether the life cycle GHG emissions mitigated across production-to-retail stages using IoT-DSL systems exceed the emissions generated by the sensors themselves. This trade-off was rigorously quantified using life cycle assessment (LCA) frameworks. Furthermore, we explore the sensitivity of emission mitigation to key parameters and highlight the leverage effects of IoT-DSL systems on climate change mitigation across food categories. By integrating scenario-based analyses, this study provides a comprehensive assessment of both immediate and long-term climate benefits. These findings deliver actionable insights to optimize IoT sensor-based technology deployment in temperature-sensitive supply chains, advancing global sustainability objectives and mitigating climate impacts.

## 2 Materials and Methods

### 2.1 Food Waste Reduction Estimation

We trace fresh-produce flows from supplier dispatch to retail sale, contrasting a static shelf life system—where discard is triggered by the printed expiry date—with our IoT sensor–driven DSL paradigm, in which real-time quality indices determine waste events (SI Section 2.1). Real-time temperature data feed a zero-order Arrhenius kinetic model (3T framework: temperature, time, tolerance; SI Section 2.2),^16,17^ parametrized via literature-derived *Q*_10_ values and activation energies (Tables S2-1–S2-7) and calibrated against 58 empirical shelf life records (RMSE ≤ 1.3 days; Tables S2–S8). Each product’s remaining shelf life is updated continuously, and removal is triggered once the predicted life falls below a predefined perishability threshold. To capture real-world variability in cold chain performance, consumer demand, and sensor accuracy, we implemented a 100-iteration Monte Carlo simulation in MATLAB R2020a (SI Section 2.1.5). In each iteration, storage temperatures are sampled uniformly within three empirically defined zones—low (0–8 °C), moderate (12–18 °C) and high (20–28 °C)—and mapped to quality-degradation rates via the temperature-coefficient method (SI Section 2.3). Retail demand is modeled over 12-h operational days, with purchase events drawn from a truncated normal distribution (mean 50 units, σ 10, truncated at 30) and evenly spaced in time; after each demand event, all items accrue 0.5 days of storage, and those with projected remaining life below 0.5 days are discarded. Initial shelf life values are similarly sampled from a truncated normal [0.3, 0.95] to reflect product heterogeneity. Each iteration tracks sold, expired, and unsold-but-safe units, and aggregating across simulations yields distributions of average remaining shelf life, waste rates, and demand-satisfaction metrics for each food category. These simulations—contextualized to Mainland China’s 2020 cold chain logistics^18^ and validated against existing case-study data^19,20^ (Table S2-9)—provide robust, data-driven estimates of avoidable waste under IoT-DSL management system.

### 2.2 Goal and Scope

This study investigated whether the life cycle GHG emissions mitigated through FLW reduction facilitated by this IoT-DSL system outweighed the additional emissions introduced by sensor deployment. It also sought to identify strategies for minimizing technological impacts while preserving the system’s climate change mitigation potential. By leveraging existing mobile Internet infrastructure for data transmission and processing, this assessment focused on the environmental trade-offs associated with integrating sensor technologies into temperature-sensitive supply chains (Figure 1). A cradle-to-retail LCA was conducted in accordance with the ISO 14044 framework^21^ and the International Reference Life Cycle Data System (ILCD) Handbook,^22^ wherein the carbon footprint is quantified by defining the goal and scope, compiling life cycle inventory data, and performing an impact assessment. The functional unit was defined as the reduction of 1 ton of FLW (“farm-to-retail”), with impacts scaled to represent China’s fresh food system. Using mass balance principles, this study accounted for the total input of fresh produce and the corresponding output, quantifying the discrepancy in food classified as waste before and after implementing this IoT-DSL system. This analysis specifically targeted the retail stage, estimating FLW reductions by calculating the shelf life extension of various food categories under optimal thermal conditions and extrapolating these benefits to upstream logistics stages. Losses due to unpredictable factors, such as mechanical damage, and byproducts were excluded to maintain focus on avoidable waste reductions. As illustrated in Figure 1, this study modeled food production, postharvest handling and packaging, cold storage, transportation, distribution, and retail (preconsumer) stages. The analyzed food categories included vegetables, fruits, meats (beef, lamb, pork, and chicken), aquatic products, dairy, and eggs. A detailed inventory analysis is provided in subsection [Sec sec2.3].

**Figure 1:**
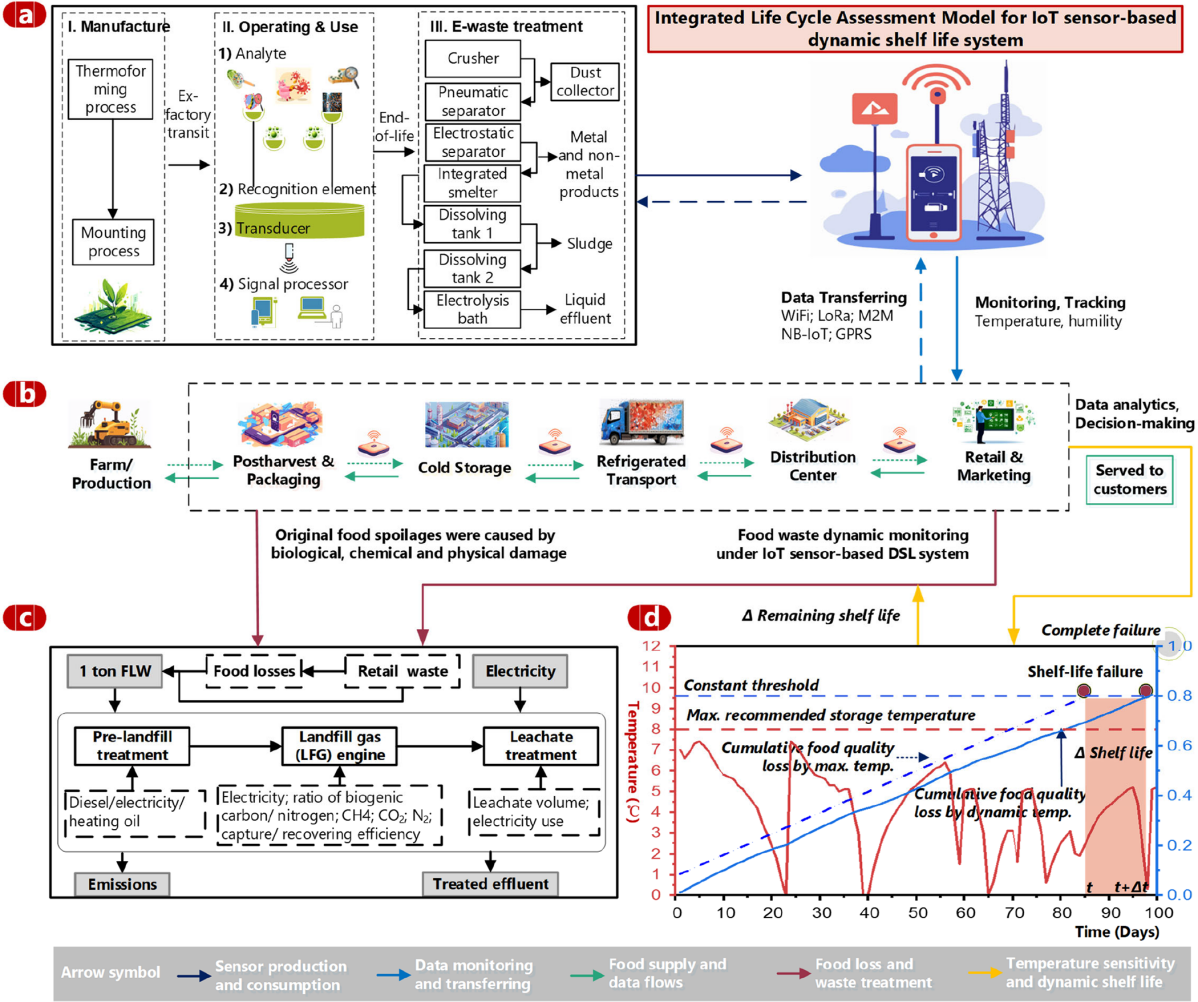
Life cycle assessment framework of IoT sensor technologies integrated with a Dynamic Shelf Life (DSL) system for food supply chains. (a) Simplified LCA analysis of humidity–temperature sensor modules, featuring environmental monitoring across supply chain stages, with data transmitted to a cloud platform via gateways and base stations, including (I) raw material input flows, (II) operational energy consumption, and (III) e-waste treatment processes. (b) Prospective LCA modeling of perishable food supply chains from farm to retail stages. (c) Food loss and waste modeling for landfill treatment scenarios. (d) Mechanism of temperature sensitivity on food quality loss uncertainties, comparing dynamic to conventional static shelf life practices for perishable food products (Icons created by https://www.freepik.com).

### 2.3 Life Cycle Inventory

This study evaluated the IoT-DSL system’s environmental impacts by analyzing selected perishable food products and IoT sensors based on LCA methodology. The life cycle inventory (LCI) was developed using a process-based modeling approach, adhering to mass balance principles. The LCIs of both food products and sensors were compiled in Sections 2.3.1 and 2.3.2, ensuring transparency and traceability in data management. Primary data sources include scientific articles, computational simulations, and publicly available databases, summarized in SI Sections 3 and 4.

#### 2.3.1 LCI Analysis of Food Supply Chains

##### 2.3.1.1 Production Stage

The production stage quantified the GHG emissions associated with agricultural activities using cradle-to-farm emission factors (EFs). These EFs, derived from peer-reviewed literature, cover diverse food categories including vegetables, fruits, meats, aquatic products, dairy, and eggs.^23–25^ The emissions reduction (EM_Pr_^food^) due to avoided FLW is calculated using eq S3-1, as detailed in Table S3-1, which outlines EFs for each food type.

##### 2.3.1.2 Postharvest Handling and Packaging Stage

Postharvest processes involved the removal of field heat and packaging to extend shelf life. The emissions from packaging (EM_Pk_^food^) are proportional to the cradle-to-gate food emissions, adjusted by the food-to-packaging ratio (FTP).^26^ Packaging-related emissions reductions are computed using eq S3-2, which attributes avoided FLW to corresponding savings in packaging burdens. The underlying EFs are detailed in Table S3-2.

##### 2.3.1.3 Cold Storage Stage

Cold storage emissions were allocated across food loss, energy consumption, and refrigerant leakage. The impact of mitigating waste (EM_St_^food^) at this stage is calculated by determining the total avoided FLW for each food category and multiplying it by the corresponding EFs specific to that category (eq S3-3). Energy consumption is calculated based on daily electricity use in refrigerated warehouses, adjusted for partial load capacities (75%). The GHG emissions (EM_ele,st_) are determined using eq S3-4, considering China’s electricity mix (carbon emission factor: 0.838 t CO_2_/MWh). Refrigerant leakage emissions (EM_leak,st_) are assessed using a 20-year equipment lifetime with annual leakage rates (8%)^16^ as detailed in eq S3-5 and Table S3-5.

##### 2.3.1.4 Refrigerated Transportation Stage

Refrigerated transportation impacts included GHG emissions from avoided food losses (EM_tr_^food^), energy use (EM_ele,tr_), and refrigerant leakage (EM_leak,tr_).^27,28^ Emissions were allocated based on the volume of food transported, specific EFs for refrigerated vehicles in China (2.63 kg CO_2_/L), and average food miles per product (eqs S3-6 to S3-8). Refrigerant leakage calculations incorporated charge quantities (3–8 kg), leakage rates (8%), and cargo turnover distances (60,000 km/year).

##### 2.3.1.5 Distribution Center

Energy consumption at distribution centers, including short-distance transportation and storage, was modeled using the same assumptions as in cold storage and refrigerated transportation stages (eqs S3-9 to S3-14). This ensured methodological consistency across supply chain stages while reflecting the energy and emissions dynamics of distribution activities.

##### 2.3.1.6 Retail Stage

At the retail stage, avoided energy consumption, refrigerant leakage, and food loss reductions were calculated based on computer-simulated results (Section 2.2). Emissions were modeled using the same approach applied in previous stages, with eqs S3-15 to S3-17 ensuring uniformity in emission calculations across the food supply chain.

##### 2.3.1.7 Waste Treatment Stage

In this study, landfill was selected as the primary treatment method for near-expired food waste, reflecting its prevalence in China. The total GHG emissions associated with this process are quantified using eq S3-18.

#### 2.3.2 LCI Analysis of Sensors

##### 2.3.2.1 Manufacturing Stage

Sensor manufacturing impacts were assessed based on their material composition, including noble metals, metals, silicon, ceramics, and plastics. A standardized global warming potential (GWP) value was applied across sensor modules due to their uniform material profiles. Emissions were calculated by multiplying GWP values by the estimated number of sensors required for each food product (eq S4-1). The nationwide sensor requirement was estimated based on temperature monitoring needs, as detailed in SI Section 4.4 and Table S4-7.

##### 2.3.2.2 Operation Stage

Operational energy consumption was calculated based on sensors’ working and sleeping states, using real-time data on voltage, current, and time distribution. Annual GHG emissions from sensors were determined using eqs S4-2 to S4-6, with emissions allocated proportionally to operating time segments. The inventory data for operational energy consumption is presented in Table S4-6.

##### 2.3.2.3 Waste Treatment Stage

End-of-life impacts of sensors were modeled to include metal recovery (e.g., Pb, Au, Zn, Ag, Cu) and residue disposal through incineration and landfill. Effluent from waste treatment was accounted for in industrial wastewater facilities. GHG emissions during the waste treatment phase were calculated using eq S4-7, highlighting the potential environmental benefits of recycling and recovery.

### 2.4 Scenarios Development

We systematically analyzed food production, perishable food demand linked to cold chain circulation rates, and energy grid dynamics for the years 2020, 2025, and 2030 to delineate multiple emission reduction pathways. Detailed data on China’s perishable food supply and demand are provided in Tables S5-1 to S5-3. The baseline scenario (BAU) was employed as a reference to assess the climate impacts of mitigating FLW in perishable food chains for 2020. For the years 2025 and 2030, we defined several targeted scenarios to explore the potential emission reduction, presented in Table 1, including:

1. **Production-side:** Increased production and cold chain circulation rate of perishable food, where the cold chain circulation rate indicates the percentage of perishable goods successfully preserved within the temperature-controlled system.
2. **Demand-side:** A 5% reduction in livestock production relative to BAU projections for 2025 and 2030, driven by shifts in China’s dietary patterns and a projected slowdown in demographic growth, reducing demand for livestock products beyond 2025.
3. **Supply side:** Improved cold chain distribution rates for perishable food by 2025 and 2030. The increase is motivated by China’s current lag in cold chain logistics compared to developed nations, which might limit the adoption of IoT-DSL systems to mitigate FLW. However, government initiatives aimed at upgrading cold chain infrastructure are expected to significantly enhance cold chain circulation rates, enabling greater technology integration and more effective FLW reduction.
4. **Energy structure:** Lowered EFs for energy units used in food refrigeration for 2025 and 2030.These are based on the projected decrease in carbon intensity of China’s electricity grid, driven by increased reliance on nonfossil energy and reduced coal usage as part of a clean energy transition.
5. **Optimized strategy:** A combination of multiple measures to achieve optimal outcomes by 2025 and 2030.

**Table 1:** Scenario Settings

scenarios	description
	2020
BAU	estimated GHG emission reductions from mitigating FLW, based on current food production, the cold chain circulation rate of perishable foods, and the energy grid structure for food refrigeration in 2020
	2025
S1	BAU scenario: Sustaining growth in food production without additional interventions relative to 2020
S2	achieving a 5% reduction in livestock production compared to 2020
S3	increasing the cold chain circulation rate for perishable foods compared to 2020
S4	reducing energy emissions intensity for food refrigeration by 20% relative to 2020
S5	implementing an integrated strategy combining interventions from S2, S3, and S4 compared to 2020
	2030
S6	BAU scenario: Sustaining growth in food production without additional interventions relative to 2020
S7	achieving a 10% reduction in livestock production based on S6
S8	increasing the cold chain circulation rate for perishable foods based on S6
S9	reducing energy emissions intensity for food refrigeration by 50% compared to S6
S10	implementing an optimized strategy combining measures from S7, S8, and S9

These scenarios enabled us to evaluate the GHG emissions reduction potential of sensor-based DSL models under varying conditions. By conducting scenario analyses, we sought to provide a nuanced understanding of how sensor technologies might contribute to emission reductions within the food chain system, depending on differing production, supply, demand, and energy dynamics.

## 3 Results

### 3.1 Unlocking Waste Reduction Potential of Temperature-Sensitive Foods

Few people are concerned about quality loss distribution of temperature-sensitive foods across various quality thresholds upon entry retail stage, which, however, is conducive to exploring the widespread impacts of enabling information technologies for specific-perishable food sectors. We assess the impact of IoT-DSL systems, outperforming static shelf life approaches in extending shelf life and reducing FLW. The findings further shed light on the capability and capacity of this IoT-DSL system on a national scale, offering a granular analysis of waste reduction contributions by food categories and supply chain stages, highlighting the potential for widespread impact. Methodologies, including system assumptions, temperature-dependent shelf life modeling, and data collection, are detailed in SI (Section 2).

Our analysis reveals that implementing sensor enabled IoT-DSL systems achieved an average extension of 1.7 to 21.8 days in shelf life across six food categories, surpassing static pathways regardless of high-, medium-, and low-quality thresholds upon retail entry (Figure 2a). Significant temporal stability enhancements are observed in specific food categories, notably meat (13.2–21.8 days) and aquatic products (14.6–21.6 days), attributed to their prolonged storage potential under frozen environment. These correspond to approximately 4-fold, 5-fold, 8-fold, and 2.5-fold as much shelf life extensions as vegetables, fruits, dairy, and eggs, highlighting significant variability driven by food type and thermal condition. The integration of IoT-DSL systems within temperature-sensitive supply chains, without accounting for external systemic adjustments, is projected to extend shelf life by 7.3–16.4% compared to conventional static ones (Figure 2b). The most significant average shelf life extensions are observed in fruits (13.8%), dairy (10.8%), and vegetables (10.0%), followed by eggs (9.4%), meat (8.6%), and aquatic foods (8.1%). These extensions varied based on quality thresholds at retail entry and the intrinsic quality heterogeneity of temperature-sensitive foods, reflecting the influence of product-specific physiological and biochemical characteristics on shelf life stabilization. Furthermore, spoilage rates for aquatic and animal-based foods accelerated significantly at medium- and low-quality thresholds, with shelf life extension rates reduced by two- to 5-fold compared to high-quality thresholds under IoT sensor deployment. This deterioration, following first-order decay kinetics, highlights the crucial need to prioritize quality control of these aquatic and animal-based foods in the early stages of refrigerated supply chains. Additionally, we assessed the influence of nontechnical factors, including consumer preferences and market demand, on food waste reduction potential. These effects are represented by the gray-shaded box plots in Figure 2b, highlighting their role in shaping waste mitigation outcomes. This study reveals mixed results for dairy products, showing a modest potential reduction in food waste rates by approximately 3.2%, despite achieving a 10.8% average shelf life extension when accounting for technical impacts on the system. While food waste reduction rates under nontechnical interventions lag behind shelf life extension rates, IoT-DSL systems achieved notable average shelf life extensions across food categories, including vegetables (7.2%), fruits (7.4%), meat (4.4%), aquatic foods (4.4%), and eggs (7.5%). These findings underscore the critical role of customer behavior in driving interconnected changes within the marketable environment.

**Figure 2:**
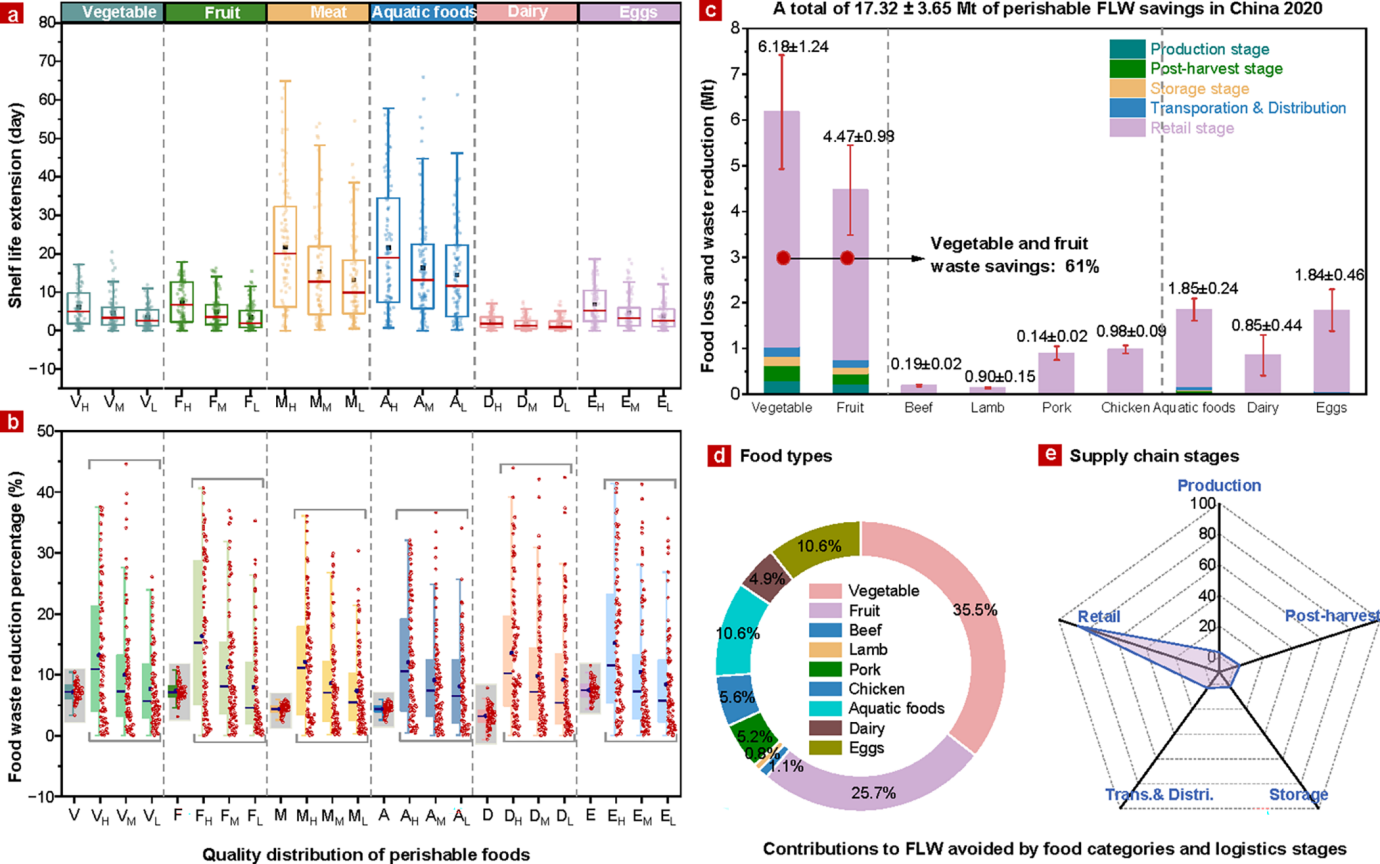
Estimated perishable food loss and waste (FLW) savings by this data-driven sensor-based IoT-DSL system in China (2020). (a,b) Box plot of shelf life extensions (days) and potential FLW reduction percentage (% per shelf life extension) across six food categories transitioning from static to dynamic expiration date approaches (Abbreviations V, V_H_, V_M_, and V_L_ on the horizontal axis denote the quality loss distribution of perishable foods under mean, highest, middle, and lowest safety thresholds upon entry into retail stages, respectively); (c) Absolute amount of FLW avoided by food categories along supply chain stages (Red bars represent root-mean-square error); (d) Contribution shares by food categories; and (e) Contribution shares by logistic stages. Note: This analysis considers only temperature-dependent food products within Chinese food systems. Calculations are based on the production volumes of each food item multiplied by their respective cold chain circulation rates.

Figure 2c demonstrates that an estimated 17.32 ± 3.65 Mt of FLW could be mitigated annually across temperature-sensitive supply chains in China with the implementation of IoT-DSL systems. Vegetables (6.18 ± 1.24 Mt) and fruits (4.47 ± 0.98 Mt) lead these reductions, collectively accounting for nearly two-thirds of the total FLW savings by mass due to their high production and consumption volumes. Pork (0.9 ± 0.15 Mt), chicken (0.98 ± 0.09 Mt), aquatic products (1.85 ± 0.24 Mt), dairy (0.85 ± 0.44 Mt), and eggs (1.84 ± 0.46 Mt) contribute 5.2%, 5.2%, 10.6%, 4.9%, and 10.6% of the total reductions, respectively (Figure 2d). In contrast, FLW reductions for beef (0.19 ± 0.22 Mt) and lamb (0.14 ± 0.22 Mt) remain minimal, comprising approximately 1% of the total due to superior supply chain practices and stringent cold chain monitoring. The hierarchical contribution to FLW avoidance spans from vegetables and fruits to aquatic products, eggs, pork, chicken, dairy, and finally beef and lamb. Moreover, Figure 2e underscores the retail sector as the critical node for FLW mitigation, accounting for substantial reductions due to its role in directly influencing consumer behavior and food disposal decisions. By leveraging IoT-DSL systems, retailers can dynamically adjust shelf life in response to real-time environmental and quality conditions, mitigating premature food disposal linked to static expiration dates. This not only reduces avoidable waste but also complements demand-side interventions such as optimized inventory management and better alignment with consumer purchasing patterns, collectively advancing the sustainability and efficiency of food supply chains.

### 3.2 Leverage of Sensor-Based IoT-DSL Systems in Life Cycle GHG Emission Mitigation

A pivotal consideration for implementing IoT-DSL systems in temperature-sensitive supply chains lies in determining whether the life cycle GHG emissions mitigated across production-to-retail stages outweigh the additional emissions introduced by the sensor modules. This trade-off is rigorously evaluated using eqs S3-1 to S3-9 and S4-1 to S4-7, with the outcomes illustrated in Figure 3.

**Figure 3:**
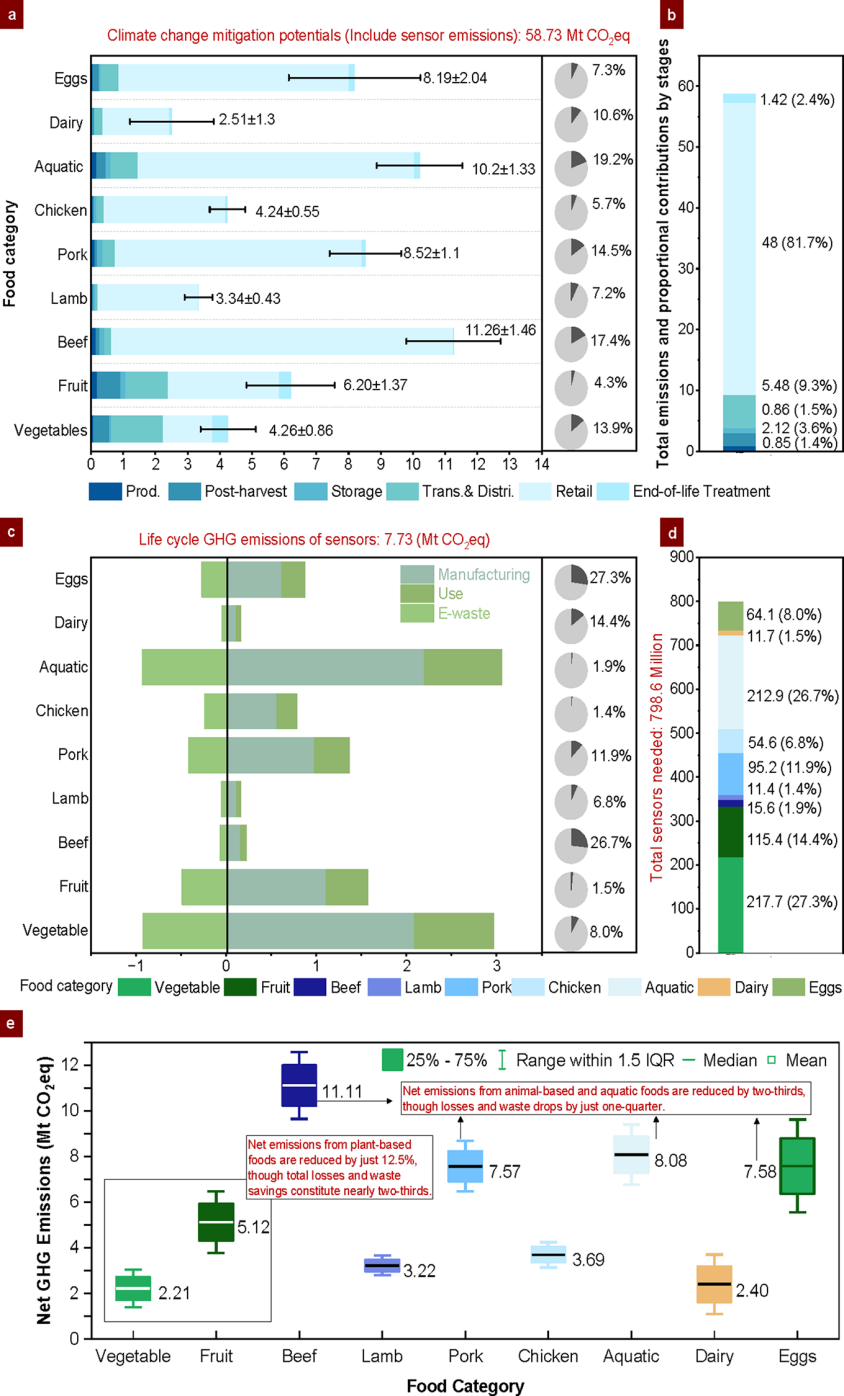
Comparison of median emissions mitigation due to FLW avoided by data-driven sensor-based IoT-DSL systems and emissions added from sensor introduction for temperature-sensitive supply chains in China (2020). The calculated net balance reflects the direct trade-off between emissions mitigated through FLW reduction and those introduced by IoT sensor deployment across each food category. (a) Absolute GHG emissions mitigated by food category along supply chain stages (Red bars represent root-mean-square error), (b) Proportional and the total emissions contributions by six supply chain stages, (c) Comprehensive life cycle climate change impacts of sensor technologies, (d) Total sensor deployment and their percentage allocations across food categories, and (e) Net environmental impacts for various food categories within China’s fresh supply chains.

The implementation of IoT-DSL systems demonstrates a significant potential to mitigate 58.73 ± 10.46 Mt CO_2_-eq of GHG emissions across food categories, with substantial variations observed by category and supply chain stage (Figure 3a). Beef emerges as the largest contributor to emissions reduction, accounting for nearly 20% of the total despite achieving only a 1.1% reduction in waste quantities. Comparable reductions are observed for pork (8.52 ± 1.11 Mt CO_2_-eq), aquatic products (10.20 ± 1.33 Mt CO_2_-eq), and eggs (8.19 ± 2.04 Mt CO_2_-eq), collectively contributing nearly half of the total GHG mitigation, even though they account for only one-fifth of total food waste reductions. These significant reductions are attributed to the high carbon intensity per unit of animal-based and aquatic products and the additional energy requirements associated with cold storage and distribution for frozen goods. Conversely, vegetables and fruits, which represent two-thirds of the total food waste reductions by mass, contribute less than one-fifth to total emissions savings. This disparity reflects their lower per-unit emission intensity, emphasizing the disproportionate relationship between food waste reduction and emissions mitigation across different food categories. Figure 3b underscores the retail stage’s dominant contribution to emission reductions (∼82%), driven by its role as the critical point of consumer interaction where the highest volumes of food waste are generated. The IoT-DSL systems thus effectively address this by dynamically extending shelf life, reducing premature disposal, and mitigating the cumulative emissions embodied in upstream processes. In contrast, upstream stages, including transportation (∼9%) and postharvest handling (∼4%), offer comparatively lower mitigation potential due to smaller FLW volumes and more refined operational efficiencies.

The GHG emissions associated with IoT sensor deployment in China’s food supply chains, as illustrated in Figure 3c, are primarily attributed to the extensive use of sensors for monitoring perishable foods. Vegetables (2.05 Mt CO_2_-eq) and aquatic products (2.12 Mt CO_2_-eq) contribute the highest emissions due to their significant distribution volumes and sensor requirements, whereas beef (0.15 Mt CO_2_-eq) and lamb (0.11 Mt CO_2_-eq) exhibit relatively lower emissions due to reduced sensor deployment needs and extended shelf lives. Sensor manufacturing is the dominant contributor, accounting for 8.0 Mt CO_2_-eq, more than twice the emissions from the use phase (3.2 Mt CO_2_-eq). Metals like gold, palladium, and silver, which account for a significant portion of sensor emissions in the manufacturing stage, could be replaced or reduced through material innovations. Moreover, improving assembly processes, such as adopting energy-efficient soldering, would further curtail manufacturing emissions. Additionally, recycling and material recovery from decommissioned sensors could mitigate up to 3.4 Mt CO_2_-eq annually, underscoring the potential of circular economy strategies in minimizing environmental burdens. The high demand for sensor modules in Figure 3d reflects the inherent complexities of China’s fresh food supply chains, where distribution volume, shelf life, and turnover rates dictate sensor requirements (Section S4.3, SI). Vegetables (27.3%) and fruits (14.4%) dominate sensor demand due to their shorter shelf lives (46 and 44 days, respectively) and high turnover rates exceeding eight cycles annually. Aquatic products (26.7%), despite their longer shelf life (180 days), require fewer turnovers but substantial sensor deployment due to their extensive distribution volumes. Variations in sensor demand are further influenced by container utilization, which is tied to food density. For instance, a 100-L container accommodates 65 kg of frozen meat but only 23 kg of vegetables or 26 kg of eggs, necessitating more containers—and thus more sensors—for lower-density foods. Conversely, dairy products, despite their short shelf life, account for just 1.5% of total sensor demand due to their high turnover rates, effectively minimizing device usage.

The distribution of net environmental impacts between avoided waste emissions by food type and added sensor emissions are displayed in Figure 3e. Without incorporating broader system changes, the IoT-DSL system delivers a net reduction in GHG emissions, mitigating 51.00 ± 10.38 Mt CO_2_-eq through avoided food waste in total, highlighting its potential as a scalable solution for emission reductions in temperature-sensitive supply chains. Beef (11.11 ± 1.46 Mt CO_2_-eq), pork (7.57 ± 1.10 Mt CO_2_-eq), aquatic products (8.08 ± 1.32 Mt CO_2_-eq), and eggs (7.58 ± 2.03 Mt CO_2_-eq) emerge as the most impactful categories in terms of emissions mitigation, with significant variability observed across these food categories. These results underscore that the GHG emissions reductions achieved through avoided food waste in these categories far exceed the emissions introduced by IoT sensors, even while achieving an approximate 25% reduction in postharvest food waste. Although vegetables and fruits constitute nearly two-thirds of total FLW savings by mass, their net emissions reduction is limited to approximately 12.5%, underscoring the leverage effect between waste reduction and emissions mitigation.

### 3.3 Sensitivity Analysis

A critical consideration for IoT-DSL systems in refrigerated supply chains is evaluating how variations in key parameters influence net emission mitigation. Figure 4 reveals that a 15% change in the emission intensity of food production (F1) drives a proportional 15% shift in net carbon mitigation, underscoring the pivotal role of agricultural emissions across food categories. This emphasizes the importance of leveraging precision technologies for real-time crop monitoring and optimizing resource inputs, such as fertilization and irrigation, to reduce agricultural carbon footprints. Other influential parameters include the shelf life of perishable foods (F2, ∼2.3%) and the capacity of standard logistics crates (M1, ∼2.7%), with 15% changes in these factors resulting in emissions shifts of 2–3%. Food transport distances (M2, ∼1.4%), natural food loss rates (F3, ∼1.1%), sensor lifetime (T2, ∼1.0%), and sensor energy consumption (T1, ∼0.9%) also contribute to emission variations but to a lesser extent. These results indicate the multifaceted nature of emission mitigation, driven by both technical factors and logistics efficiency. Although parameters such as T3 and M4 exhibit minimal sensitivity in this analysis, their importance may emerge under different system boundaries or objectives, illustrating the complexity of evaluating supply chain technologies. This analysis highlights the need for context-specific assessments to optimize IoT-DSL systems and ensure robust emission reductions in refrigerated supply chains. Addressing these critical parameters could help maximize the environmental benefits of deploying digital technologies across temperature-sensitive food systems.

**Figure 4:**
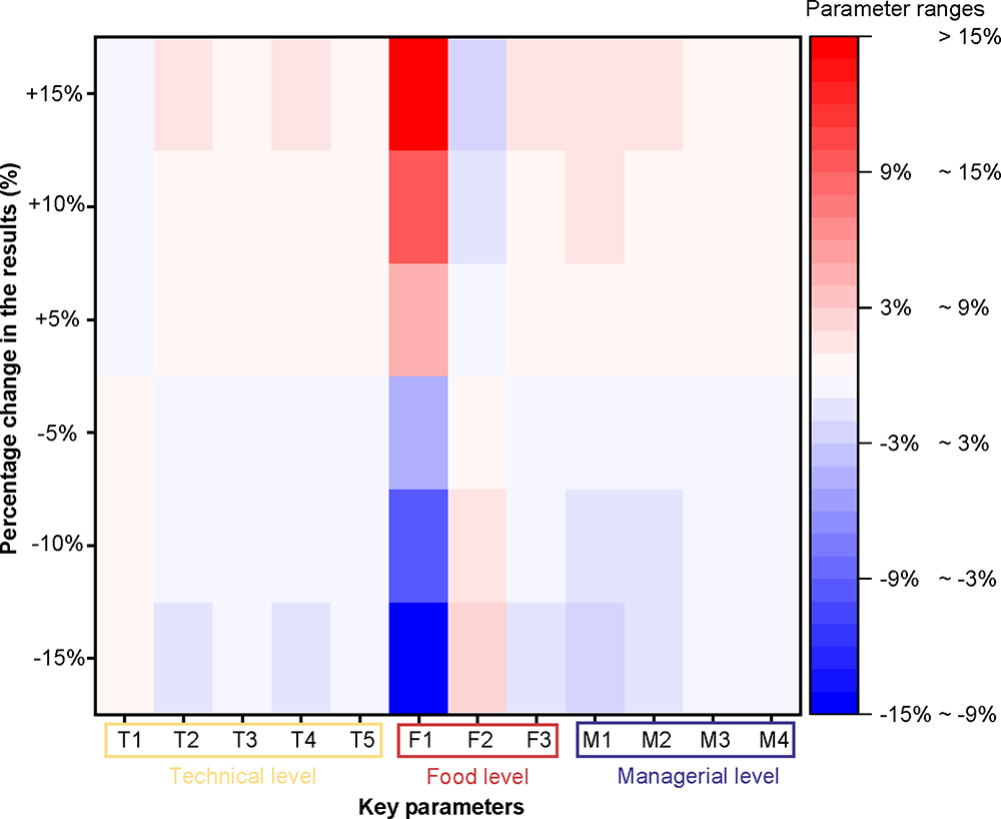
Sensitivity Analysis. A one-factor-at-a-time (OFAT) approach was employed to assess the sensitivity of emissions reductions from avoided FLW to critical parameters in the life cycle inventory (LCI) modeling. Each parameter was held constant at its average value, while the parameter of interest was independently adjusted by ± 5%, ± 10%, and ± 15%. Key assumptions influencing emissions reductions were categorized into three aspects: technical factors, food-specific factors, and managerial factors. Parameters on the horizontal axis represent: T1, energy consumption of sensors; T2, sensor lifetime; T3, emission factors for packaging; T4, emission intensity of the sensor recovery; T5, emission intensity of the cold storage; F1, emission intensity of food production; F2, shelf life of fresh food; F3, natural loss rate of food; M1, capacity of standard logistics crates; M2, food transport distances; M3, cold storage duration; and M4, refrigerant leakage rate.

### 3.4 Scenario-Based Projections and GHG Emissions Mitigation Strategies

The leverage effects of IoT-DSL systems on climate change within China’s food supply chains exhibit significant variability across food categories. This variability likely arises from factors such as differences in production capacity, supply chain enhancements, demand-side adjustments, and energy transitions. Access to refrigeration substantially influences food demand and dietary patterns as nations develop, emphasizing the critical role of robust refrigerated supply chains in facilitating these shifts.^29^ The effectiveness of IoT-DSL systems in extending shelf life and mitigating FLW is further enhanced in well-functioning cold chain environments.^1^ As a result, the net avoidable emissions for each food category are assessed relative to BAU levels, highlighting their potential contributions to carbon mitigation efforts.

While carbon mitigation potential varies significantly by food type and scenario, Figure 5a illustrates that IoT-DSL systems achieve 1- to 4-fold and 0.5- to 1.5-fold increases in emissions reductions compared to BAU levels in 2020 for the vegetable and fruit sectors, respectively. These improvements stem from enhanced sensor deployment in refrigerated supply chains, enabling better monitoring of perishable vegetables and fruits, particularly as advancements in cold chain circulation rates help reduce emissions. Food types exhibiting the greatest carbon mitigation potential are beef, pork, and aquatic products. For example, beef demonstrates substantial reductions of 16.68 Mt CO_2_-eq (S3) and 18.95 Mt CO_2_-eq (S8), representing growth rates of 50.1% and 70.6% in emission mitigation under scenarios emphasizing technological progress. These advancements enable wider deployment of IoT-DSL systems across cold chains for beef products, facilitating greater reductions in emissions. However, mixed results are observed for meat, aquatic, and other animal-based products compared to plant-based foods under scenarios such as S3 and S8. While net carbon mitigation remains higher in these scenarios than in optimized strategies (such as S5 and S10), the observed variability highlights the critical role of dietary shifts. Reduced meat consumption, driven by its significantly higher carbon intensity per unit, unlocks greater mitigation potential. This underscores the interplay between technological adoption, dietary behavior, and emissions outcomes in food systems.

**Figure 5:**
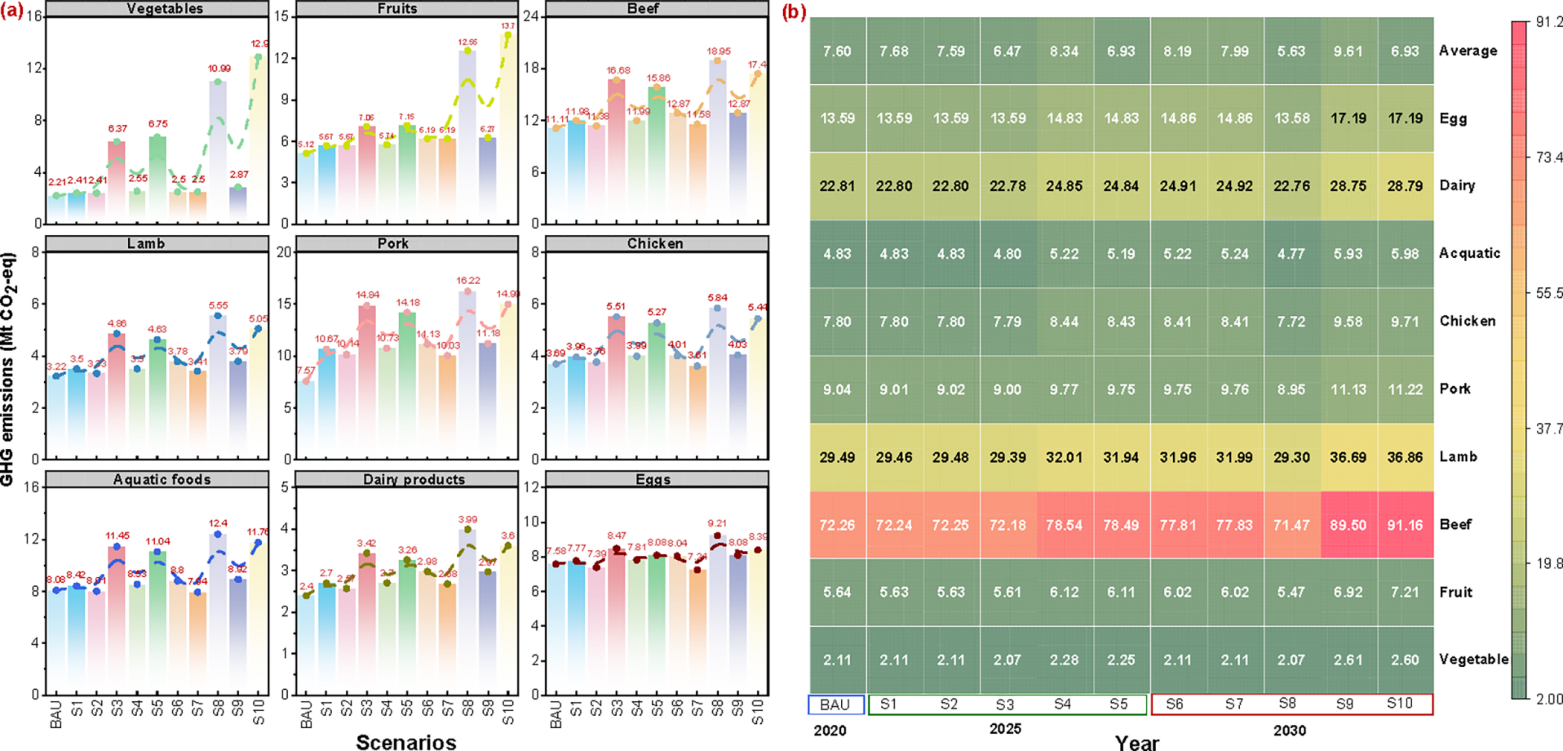
Net climate change mitigation potential and resource use efficiency assessment for different food categories from 2020 to 2030 across multiple scenarios. Panel (a): Histograms illustrating net avoidable GHG emission impacts for individual food categories, including vegetables, fruits, beef, lamb, pork, chicken, aquatic foods, dairy products, and eggs. Panel (b): Heatmap showing resource efficiency indicators for sensor deployment in temperature-sensitive supply chains, evaluated by food category and year (2020, 2025, and 2030). The indicator is derived by dividing the GHG emissions avoided due to FLW reductions enabled by the IoT sensor-based DSL system by the annual GHG emissions generated by the life cycle of sensors themselves. Scenarios include advancements in cold chain circulation rates, optimized production strategies, reduced livestock demand, improved logistics, and cleaner energy profiles. Detailed scenario settings and supporting data are provided in Tables 1 and S5-1 to S5-3.

We applied *Input-Output Efficiency* (IOE) indicators to assess whether the carbon mitigation achieved through FLW reduction offsets the life cycle impacts of IoT sensors deployed in temperature-sensitive supply chains. As shown in Figure 5b, the average IOE value for all food categories is estimated at 17.5 in 2020, indicating that for every unit of GHG emissions generated by sensors, 17.5 units of GHG emissions from food production and consumption are mitigated. Beef exhibits the highest mitigation efficiency, with an IOE value of approximately 72.3 in 2020, followed by lamb (29.5) and dairy products (22.8). In contrast, vegetables (2.1), fruits (5.6), chicken (7.8), aquatic products (4.8), and eggs (13.6) show relatively lower IOE value. These results reveal a strong correlation between the IOE value of sensors and the carbon emission intensity per unit of product, emphasizing the potential of IoT sensor technologies to drive disproportionate benefits in high-emission food categories. Moreover, the environmental benefits of IoT-DSL systems are projected to increase steadily over time. For instance, the IOE value for the entire food system is estimated to rise to 18.9 by 2030, up from 17.5 in 2020. This improvement is largely attributed to evolving dietary patterns, including a higher share of meat products (e.g., beef) and a relative decline in fruits and vegetables. Concurrently, advancements in China’s energy matrix, characterized by a growing reliance on renewable energy, will further lower carbon emissions associated with food refrigeration. The expanding demand for perishable foods within cold chain logistics is anticipated to drive the wider adoption of IoT sensor technologies. The accelerated development of cold chain infrastructure and IoT-enabled solutions is expected to enhance the IOE of sensors, thereby fostering a transition to a low-carbon food system. However, a declining trend in the IOE is observed as cold chain distribution rates increase across food types (e.g., scenarios from S3 to S8). This is primarily due to the static shelf life assigned by suppliers and printed expiration dates on food packages, which remain largely unchanged under highly optimized refrigerated supply chains. In such scenarios, the dynamic shelf life aligns closely with the static shelf life, reducing the net environmental benefit per unit of sensor applied, despite improvements in cold chain management practices.

## 4 Discussion

The use of IoT sensors has been recognized for over a decade as a promising tool to enhance sustainability in food systems, providing real-time insights that drive measurable reductions in FLW.^6,30^ However, significant gaps remain in quantifying their environmental trade-offs, particularly as technological advancements in food systems lag behind other sectors, especially in developing economies.^31^ This study highlights the nuanced trade-offs between the environmental impacts of IoT sensors and the climate mitigation benefits achieved through FLW reduction. A self-coding quality loss model was employed to dynamically calculate the shelf life of perishable foods, enabling a more precise evaluation of FLW reduction rates under IoT sensor-based DSL systems compared to static use-by-date methods. Quantitatively, the volume of avoided losses was directly proportional to the scale of food provision in each category, with larger-supply categories yielding the greatest absolute reductions in waste. Our estimates suggest that, by targeting China’s temperature-sensitive supply chains for cold-chain–dependent categories, this strategy could avert some 17.32 ± 3.65 Mt of FLW each year. However, the magnitude of these climate mitigation potentials varies significantly across food categories and life cycle stages. Collectively, this innovative system could yield a net GHG emissions reduction of 51.00 ± 10.38 Mt CO_2_-eq annually, equivalent to 11% of China’s total FLW-related emissions (464 Mt CO_2_-eq).^32^ Beef, despite achieving only a 1.1% reduction in waste, delivers the largest emissions benefit—nearly 20% of the total—while pork, seafood and eggs collectively account for almost half of all GHG savings, even though they represent just one-fifth of the mass-based waste reductions. In contrast, vegetables and fruits—which comprise two-thirds of the avoided waste by weight—contribute under 20% of total emissions abatement, underscoring the stark disparity between mass-based waste reductions and carbon-mitigation potential across food categories. The broader implications of deploying IoT sensor-based DSL systems, including potential unintended consequences, must be carefully considered.^33^ Notably, sensor fabrication during the manufacturing phase imposes disproportionately high environmental burdens relative to other life cycle stages. Overcoming this demands the development of greener materials—such as soluble conductors and degradable polymer matrices—and the enhancement of e-waste recovery systems, both of which are essential to fully unlock the climate-mitigation potential of IoT-enabled, temperature-sensitive supply chains.

The proposed systems deliver 8.1–13.8% shelf life extensions across fresh foods—peaking in fruits, dairy, and vegetables—yet these gains shrink two- to 5-fold for aquatic and animal products at lower quality thresholds, underscoring the need for early chain quality control. By comparison, nontechnical drivers yield just 3.2–6.5% waste reductions, highlighting the necessity of pairing technical and behavioral strategies. According to the World Economic Forum,^33^ food waste could decline by 5–7% by 2030 with 50–70% market penetration of food-sensing technologies in industrialized regions. Other studies have similarly demonstrated the sustainability benefits of innovative technologies. RFID systems, for example, reduce fresh milk waste by approximately 2.6% across its life cycle through enhanced inventory management.^34^ Our findings indicate a (3.43 ± 1.78) % reduction in milk waste attributable to the IoT-DSL system, marginally surpassing the savings potential achieved by RFID technology. Simultaneously, the environmental benefits of waste reduction offset the lifecycle impacts of RFID technology more than 5-fold, a value that could increase to 7.5-fold with smart sensors in our analysis. Moreover, the use of expiry-date indicators, such as the keep-it system, has been shown to reduce fish waste by 3.2%, saving 0.1 kg of food and preventing 0.44 kg CO_2_-eq emissions per EUR invested. Our modeling estimates aquatic product waste reduction at (4.14 ± 0.54)%, closely aligning with Lehn et al. (2023).^35^ Although Zhu et al. (2022) project 20–41% reductions in food loss under large-scale IoT sensor roll-out,^4^ the variability in these high-level estimates belies important differences in scope and methodology. Unlike the Luo et al. Perspective—which conceptually explored IoT’s role in long-chain products,^30^ applied literature-derived FLW rates (i.e., 8–60%) and assumed unfettered cold-chain support—our study narrows its scope to key food categories (vegetables, fruits, meat, aquatic products, dairy and eggs), embeds sensors within specific application systems, and explicitly models cold-chain circulation as a binding constraint. Further, by replacing second-hand reduction rates with a bottom-up, first-principles framework calibrated to food temperature sensitivity, circulation rates and sensor performance, we generate realistic, context-sensitive FLW estimates. These methodological advances reveal how technical efficacy, infrastructure maturity and product heterogeneity converge to shape actual waste-reduction outcomes—and provide a roadmap for targeted investment in sensor-driven mitigation strategies. Our results underscore the transformative power of digital, sensor-enabled interventions to curb waste and emissions across agri-food chains; however, real-world uptake remains nascent—particularly in emerging economies—mandating targeted investment, policy frameworks, and capacity-building to bridge this implementation divide and unlock their full sustainability potential.

Our study focuses on the potential of IoT sensor technology in enhancing the climate change mitigation potential of temperature-sensitive food systems. However, a critical question remains: could the adoption of such technologies offer meaningful environmental benefits for noncold chain foods? Addressing this question requires future research to evaluate the scalability and applicability of IoT sensors across broader food system contexts. Moreover, expanding the environmental analysis beyond GHG emissions to include land use, water stress, acidification, and ozone depletion is crucial for a more comprehensive sustainability assessment. An additional challenge lies in the potential risk of exacerbating inequality in access to such technologies due to the digital divide, particularly in developing countries. Considering that micro, small, and medium-sized enterprises dominate food supply chains, it is imperative to thoroughly assess the economic costs and benefits of scaling up these technologies. We recognize that, despite our detailed consumer-behavior modeling, empirical validation of end-user trust remains an important next step. Consumer surveys indicate that freshness extension and waste reduction are high priorities for shoppers,^36^ suggesting strong market receptivity once benefits are clearly communicated. Moreover. field pilot studies—using in-store signage, app-based feedback, and postpurchase surveys—will be needed to translate these insights into quantifiable measures of user trust and willingness to pay.^37^ Therefore, striking a balance between affordability and the environmental and operational advantages will be critical to fostering equitable adoption and maximizing the sustainability potential of IoT sensor systems in global food systems.

## Supplementary Material



## Data Availability

The MATLAB programming code and computation scripts are available upon request.

## Notes

Evidence and detailed data associated with this article (PDF)
