# Supplementary material for: Reducing Food Loss and Associated Greenhouse Gas Emissions Using a Dynamic Shelf Life Approach
Source: Environ Sci Technol. 2025 Jun 30;59(27):13742–53. doi: 10.1021/acs.est.5c04093 (PMC12269072; doi:10.1021/acs.est.5c04093)
Supplement: Supplementary file 1 [file es5c04093_si_001.pdf]

Supporting Information

Reducing Food Loss and Associated Greenhouse Gas Emissions Using a Dynamic Shelf Life Approach

Junzhang Wu[a], Yifeng Zou[b], Gang Liu[c], Li Xue[d, e], Zhimin Shi[d], Andrea Fedele[a], Alessandro Manzardo[a, *]

[a] CESQA (Quality and Environmental Research Center), Department of Civil, Environmental and Architectural Engineering, University of Padova, Via Marzolo 9, 35131 Padova, Italy

[b] School of Management, Guangzhou University, 510006, Guangzhou, China

[c] College of Urban and Environmental Sciences, Peking University, 100871, Beijing, China

[d] College of Economics and Management, China Agricultural University, 100083, Beijing, China

[e] Academy of Global Food Economics and Policy, China Agricultural University, 100083, Beijing, China

[f] State Key Joint Laboratory of Environmental Simulation and Pollution Control, School of Environment, Beijing Normal University, Beijing, 100875, China

---

Number of Pages: 65

Number of Figures: 9

Number of Tables: 35

* **Corresponding Author:** Alessandro Manzardo — *CESQA* (Quality and Environmental Research Center), Department of Civil, Environmental and Architectural Engineering, University of Padova, Via Marzolo 9, 35131 Padova, Italy; Email: alessandro.manzardo@unipd.it

Figure and Table of Contents

Figure S1-1: WoS–sourced studies identified and screened 8

Figure S1-2: Annual publication counts (2003–2022) 10

Figure S1-3: Publications by journal type 11

Figure S1-4: Publication share by food group 12

Figure S1-5: Publication share by supply-chain stage 13

Figure S1-6: Global publication distribution (world map) 14

Table S1-1: Keyword clusters and counts 15

Table S1-2: Top-30 keywords by occurrence 16

Figure S1-7: Keyword co-occurrence network (VOSviewer) 17

Figure S2-1: FSL vs. DSL system framework for temperature-sensitive foods 22

Table S2-1: List of 58 selected fresh foods 28

Table S2-2: Fruit cold-chain temperature requirements 30

Table S2-3: Vegetable cold-chain temperature requirements 31

Table S2-4: Aquatic-product temperature requirements 32

Table S2-5: Livestock and poultry meat temperature requirements 32

Table S-6: Other fresh-food temperature requirements 32

Table S2-7: Retail shelf temperatures for fresh foods 33

Table S2-8: Statistical performance in refrigerated ranges 35

Table S2-9: Avoided food waste rate along the FSC. 36

Table S3-1: Cradle-to-gate emissions factors for different perishable foods 37

Table S3-2: Packaging emissions per food category 38

Table S3-3: China's energy-mix share 40

Table S3-4: Cold-storage emissions allocation data 40

Table S3-5: Refrigerant-leakage allocation data 41

Table S3-6: Refrigerated-transport emissions allocation data 42

Table S3-7: Distribution-phase emissions allocation data 44

Table S3-8: Temporary-storage emissions allocation data 44

Table S3-9: Retail-phase emissions allocation data 46

Table S3-10: Retail display-case energy consumption 46

Table S3-11: Bulk densities of food products 46

Table S3-12: Non-refrigerated energy allocation 47

Table S3-13: Landfill-calculation inputs for 1 t food waste 47

Figure S4-1: Food-sensor system boundaries 52
Table S4-1: Sensor module components (fruits, vegetables, dairy) [6] 52
Table S4-2: Sensor module components (meat, fish, eggs) 52
Table S4-3: LCI of humidity-temperature sensor raw materials 53
Table S4-4: Sensor and component electricity consumption 55
Table S4-5: LCI of sensor-module treatment and recycling 56
Table S4-6: Estimated sensor counts per food type 58
Table S4-7: Intelligent container design specifications 58
Table S4-8: Food density (weight per unit volume) 58
Table S5-1: 2020 food production, circulation, demand (China) 60
Table S5-2: 2025 food production, circulation, demand (China) 60
Table S5-3: 2030 food production, circulation, demand (China) 61

1 Literature Review

1.1 Background and Purpose

The need for a swift transition to an environmentally sustainable food system is pressing. [1,2] If technology and behaviors in the food system persist along recent trajectories, international climate and biodiversity targets would then elude us in the coming decades. While the socio-economic consequences, such as increased public costs, labor demand, and food prices, are easily observable in everyday life, the environmental impacts associated with food loss and waste (FLW) pose a challenge as they remain hidden and are not immediately recognizable by companies, consumers, and governments. [3,4] A crucial stride toward achieving international and regional targets for reducing FLW is to evaluate and then forecast the potential of sensor-based solutions leveraging digital technologies, facilitating the transition toward an environmentally sustainable food system.

Industry 4.0 comprises a wide range of cutting-edge digital technologies and solutions, including robots, the Internet of Things (IoT), artificial intelligence (AI), and smart sensors. These developments have the potential to hasten automation and the uptake of digital practices across a range of industrial sectors, including the food sector. The integration of the IoT into food sectors offers several advantages, including better refrigeration, real-time quality monitoring, optimized delivery routes, and improved distribution methods. Recent advancements in food supply chain optimization primarily revolve around extending the shelf life of perishable items and reducing FLW during the storage or distribution process. Practical examples have been successfully applied to various perishable foods, enabling real-time management and predictive capabilities for extending shelf life and decreasing FLW. [5] The introduction of IoT has brought about significant transformations and complexities within the food supply chain, impacting both the supply chain itself and environment impacts. It is estimated that

applying IoT and sensor technologies in 50–75% of supply chains in developed nations by 2030 could lead to a reduction of food loss by 10–50 million tons during distribution, highlighting environmental benefits caused associated with FLW prevention and mitigation. [6] However, only a limited number of individuals express concern regarding the environmental sustainability implications of the widespread adoption of IoT. It is crucial to account for the environmental challenges associated with sensor manufacturing, operation, and electronic waste disposal that arise with the widespread adoption of IoT since this is conducive to directing the development of design and leveraging enabling technologies in an environmentally beneficial way, particularly in the context of food-specific sensor modules. Therefore, considering potential life cycle trade-offs in relation to the integration of IoT into the food supply chain is necessary. [5,6]

The main purpose of the research is to perform a comprehensive and systematic review of the literature, focusing on the utilization and outcomes of IoT sensor technology within food supply chains. Our emphasis is on its capacity to prolong the shelf life of food products and reduce FLW across the entire value-added chain. Additionally, our analysis aims to evaluate the ramifications of incorporating IoT sensor technology on environmental dimension, adopting a Life Cycle Assessment (LCA) perspective. The findings provide comprehensive insights for guiding the implementation of data-driven IoT sensor-enabled technologies in the food supply chain. Particularly, this analysis can assist in informing and shaping effective food waste policies and strategies through the utilization of such advanced technologies.

1.2 Gaps and Challenges

The application of food sensing technologies, rooted on the IoT, holds significant promise for revolutionizing food supply chains. [7] This emerging field offers opportunities for precision, traceability, visibility, and controllability. The exponential growth of sensor-based solutions in reducing FLW also presents a vast potential for

generating valuable data in this context. Despite the increasing interest in food sensing technologies within the IoT application domain, their potential to effectively and sustainably reduce FLW remains questionable. Firstly, while the IoT-based technologies theoretically enable real-time tracking and data sharing, improving communication in the food supply chain, the main characteristics of food sectors from "farm-to-fork" in the context of the IoT domain still require empirical validation. Variations in food types, technology usage, geographical locations, research methodologies, and other factors across studies introduce challenges in comprehending the actual impact of these technologies. This underscores the need for a comprehensive review of all pertinent studies to identify disparities and commonalities, thereby providing guidance for future research studies. Secondly, the scarcity of knowledge regarding the utilization of IoT sensor-based real-time monitoring technologies within the food industry, along with the associated practices, underscores the necessity for a meticulous and systematic exploration of the potential applications of intelligent monitoring technologies to tackle issues within the food supply chain. A commonly accepted approach to address this need is the evaluation of the impact of these technologies on extending shelf life and reducing FLW across the value-added chain. Thirdly, in addition to understanding penetration of sensor technologies into the food supply chain and its uses to solve possible problems, the integration of these technologies necessitates an exploration of potential life cycle trade-offs, with a particular focus on environmental impacts like carbon mitigation. [5] However, studies employing LCA to assess these trade-offs are exceedingly limited. Consequently, there is an imperative to scrutinize existing research and highlight the demand for additional investigations in this area.

1.3 Objectives of Literature Review

The objective of this analysis is to conduct an extensive and systematic literature review to analyze the use and effects of IoT in food supply chains, with an emphasis on its

potential to extend the shelf life of food products and decrease FLW across the entire value-added chain. Meanwhile, our study aims to examine the impact of IoT sensor technology integration on environmental, economic, and social aspects from an LCA perspective. It seeks to assess whether the potential adverse effects stemming from technological production and waste outweigh the environmental advantages associated with avoided FLW, as well as the extension of shelf life.

1.4 Methods of Literature Review

Search strategy and study selection. Our review was conducted following PRISMA (Preferred Reporting Items for Systematic Reviews and Meta-Analyses). As shown in **Figure S1-1**, Eligible studies were identified by conducting a comprehensive keyword search on the Web of Science (WoS) databases in a Boolean operation, i.e., Title, Abstract, Keyword = "food waste" OR "food loss" AND “sensor” AND "dynamic" OR “real-time” OR "internet of things" OR "IoT". We then met our inclusion criteria for the literature search: the original article and review article, written in English and published online from 2003 to 2022. In total, there were 791 articles available on WoS. Subsequently, six hundred and thirty-nine (n=639) records from the literature review database were removed because of being out of the research scope. We further excluded papers (n=81) that have no relation to FLW via real-time IoT-based technology from a life cycle assessment (LCA) perspective by browsing the title and abstracts. In this way, we retrieved 71 papers to explore the role of real-time sensor-based technologies in reducing FLW along the value-added chain.

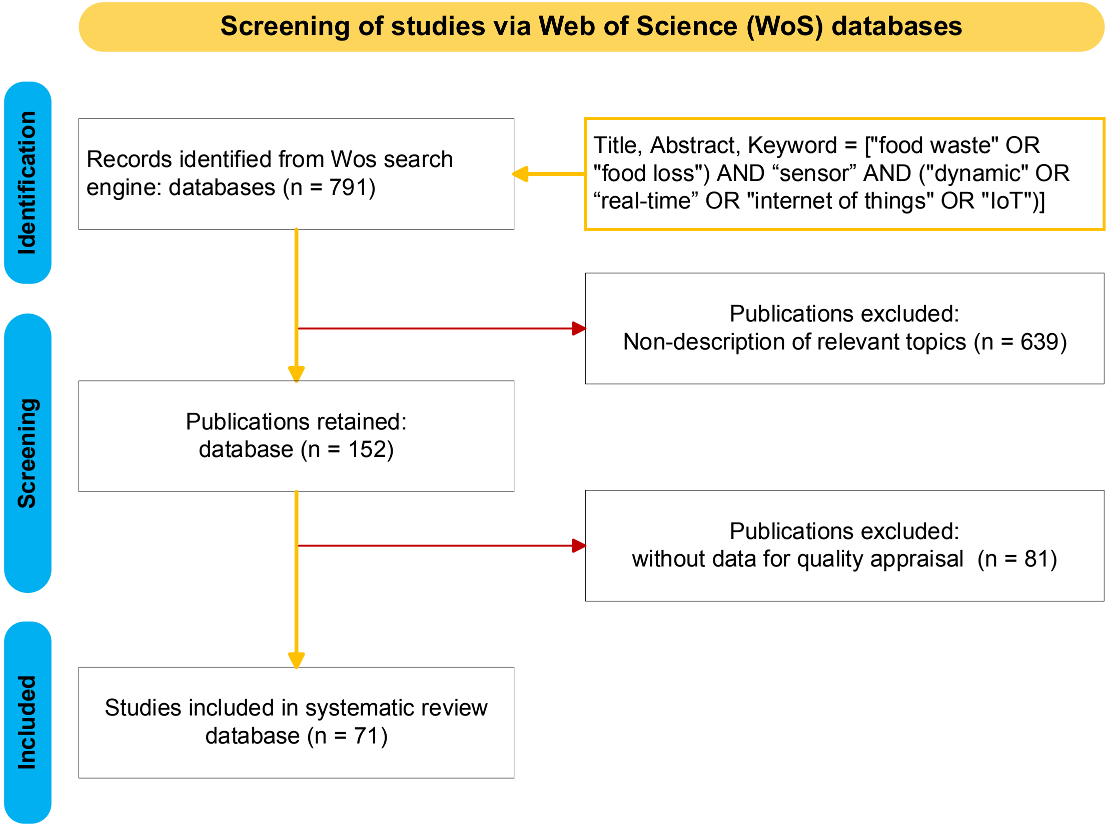

Figure S1-1: WoS–sourced studies identified and screened

Quality Appraisal. While the exact terminology to describe IoT sensor-related technologies varies under different scenarios, this analysis highlighted crucial potentials, gaps between previous studies, and future trends by using such technologies in the food supply chain as found in the literature. The application of IoT holds significant promise for revolutionizing food supply chains by offering opportunities for precision, traceability, visibility, and controllability. The exponential growth of IoT sensor-based solutions in reducing FLW also presents a vast potential for generating valuable data in this context. For example, inefficient stock rotation of products with a shorter remaining shelf life can lead to losses in storage or retail stores. As such, innovative IoT-based technologies can facilitate FLW reduction across the entire value-added chain in food systems.

Data Extraction and Analysis. The relevant articles identified in this review were

created from WoS database and then analyzed using Google Sheets. The literature review was conducted based on the inclusion criteria of extending the shelf life of food products to prevent FLW via real-time technologies in the context of the IoT domain from an LCA perspective. Furthermore, this study used VOSviewer version 1.6.19 that offers text mining functionality to construct and visualize co-occurrence networks of important terms extracted from a body of scientific literature. Co-occurrence networks can be generated using data obtained from the WoS, enabling the identification of relationships and interactions among various subject areas. Network visualization includes multidimensional scaling and clustering features, making it a potent method for analyzing diverse bibliometric networks, such as keyword relationships. In network visualization, clusters are color-coded to represent specific node properties. For example, nodes may signify keywords, and node size can indicate the frequency of keyword citations. Terms that co-occur frequently tend to be positioned closely to each other in the visualization.

1.5 Result Analysis of Literature Review

Status Quo of IoT Technologies in the Food Supply Chain. The retrieved 71 papers used for describing the contribution of IoT technologies to mitigate FLW along the value-added chain can be categorized by years of publication, types of publication, food categories and stages. The number of articles published between the years of 2003 and 2022 is analyzed to identify trends and patterns in articles with the intention of shedding light on the development of research in this field. **Figure S1-2** shows how articles are distributed throughout these two decades. Notably, there were no publications noted until the year 2008. Thus, this first level highlights the preliminary phases of investigation and study of IoT sensor applications in relation to FLW along food supply chains. Beginning in 2008, there was a noticeable change as publications started to rise. This significant surge from 2014 onwards points to a time of increased research effort and the top of this rising trend, which included 30 articles, was reached in 2022. The

discovered patterns show the increasing interest in the use of IoT applications in FLW mitigation along food supply chains. The initial years saw limited interest, possibly due to the nascent nature of IoT technology and its application in the food industry. However, there was a noticeable rise in research production starting in 2014, demonstrating a greater understanding of the potential advantages of IoT in reducing FLW within the food supply chain.

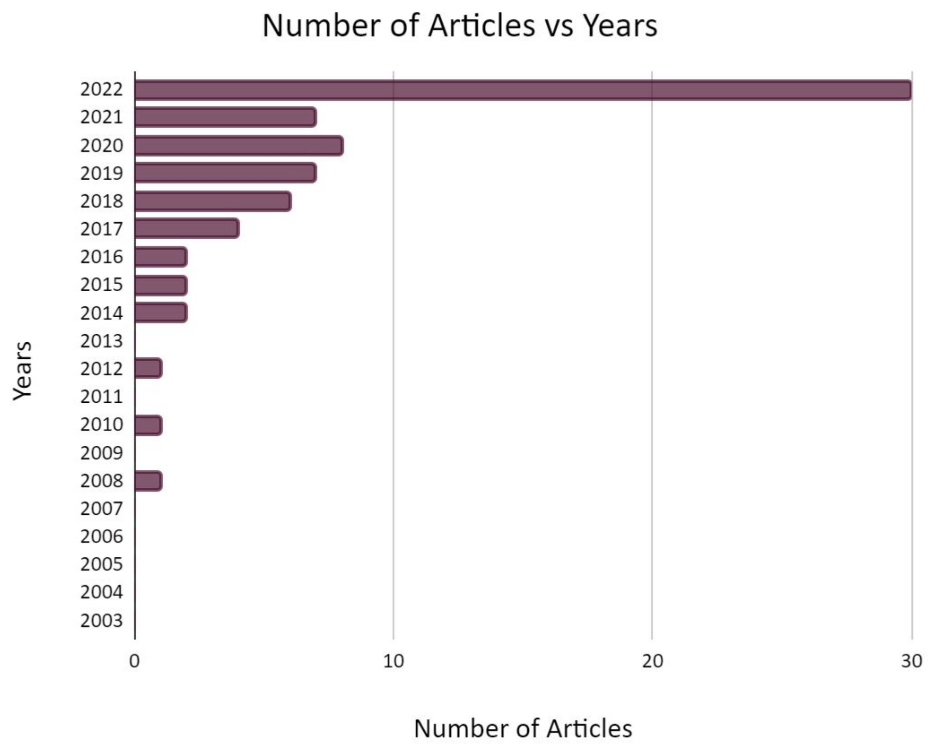

Figure S1-2: Annual publication counts (2003–2022)

Figure S1-3 demonstrates the number publications on IoT sensor in reducing FLW along food supply chains published in different journals. With seven articles in the lead, "Sensors" highlights its crucial function while with six articles, "Food Control" follows immediately. The journal "Foods" makes a strong showing with four articles. Moreover, two publications from each of many other journals which are "Comput. Electron. Agric.," "Comput. & Ind. Engineering," "Food Chemistry," "Food Hydrocolloids," "Food Packag. Shelf Life," "IEEE Access," "J. Food Eng.," "J. of Cleaner Production,"

"Procedia Comput. Sci.," and "Sustainability," are included. The interest in IoT applications in reducing food supply chains worldwide is reflected in a variety of other journals and this variety highlights the need for multidisciplinary collaboration.

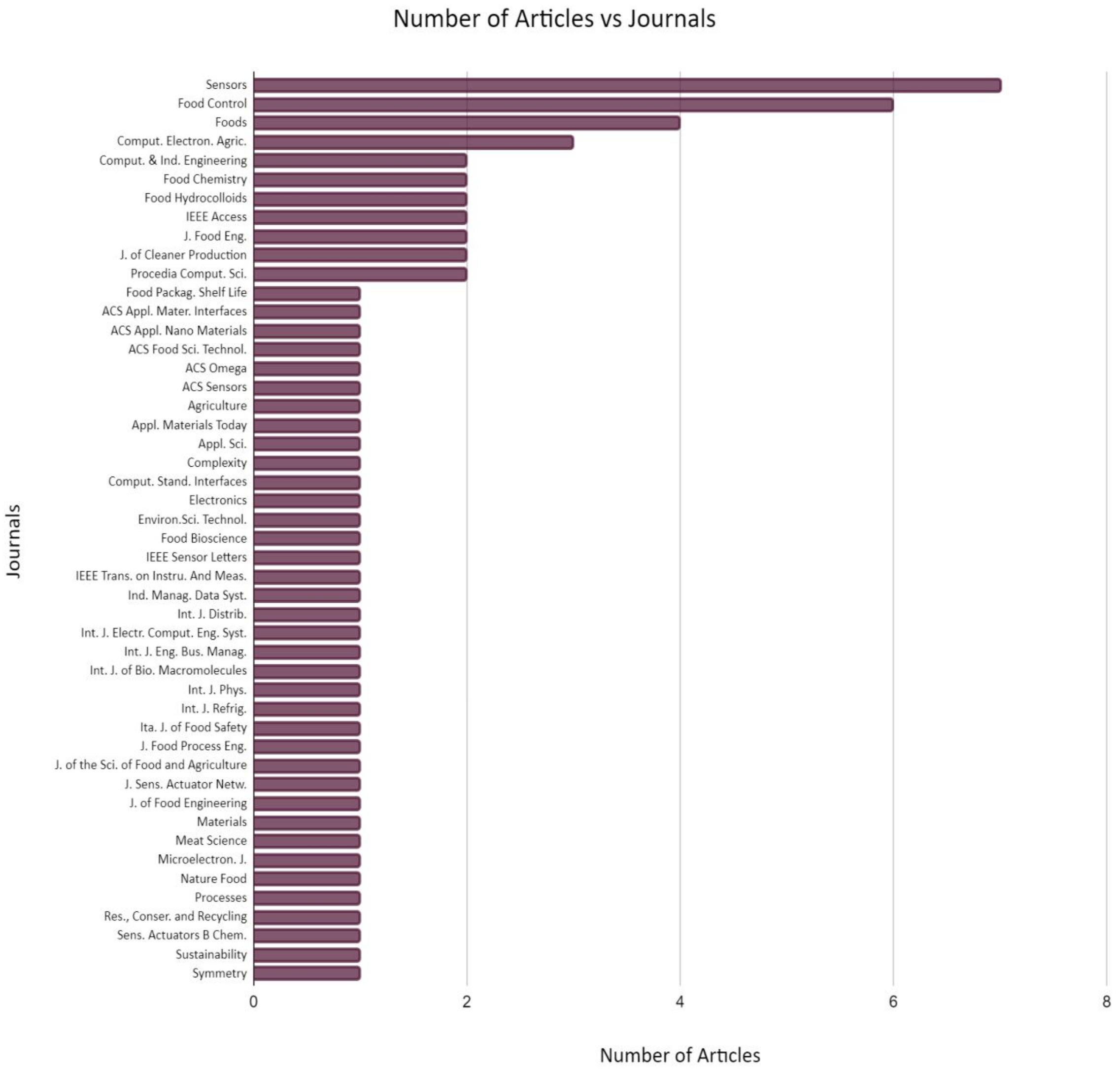

Figure S1-3: Publications by journal type

Figure S1-4 shows the percentage of articles based on different food groups. With 25,4%, papers devoted to examining IoT applications in mitigating FLW across multiple food groups, evaluating more than one food group in each study appears to be a popular choice. 16.9% of papers examine fruits which come in second place in terms of research concentration while with 15.5% of publications, meat and fish appear as

other significant food categories. This indicates a considerable interest in utilizing IoT to improve the resilience of the fruit, meat and fish supply chain. Vegetables, with 8.5% of articles, represent another important focus group. Furthermore, smaller numbers of entries for Cereals, N/A (representing unidentified food categories), Beverages, Composite Dishes, roots, tubers, plantains, and spices and condiments also exist. This reveals that focus on non-perishable food groups are less than perishables while revealing the adaptability of IoT technology in reducing FLW across different food supply chains simultaneously.

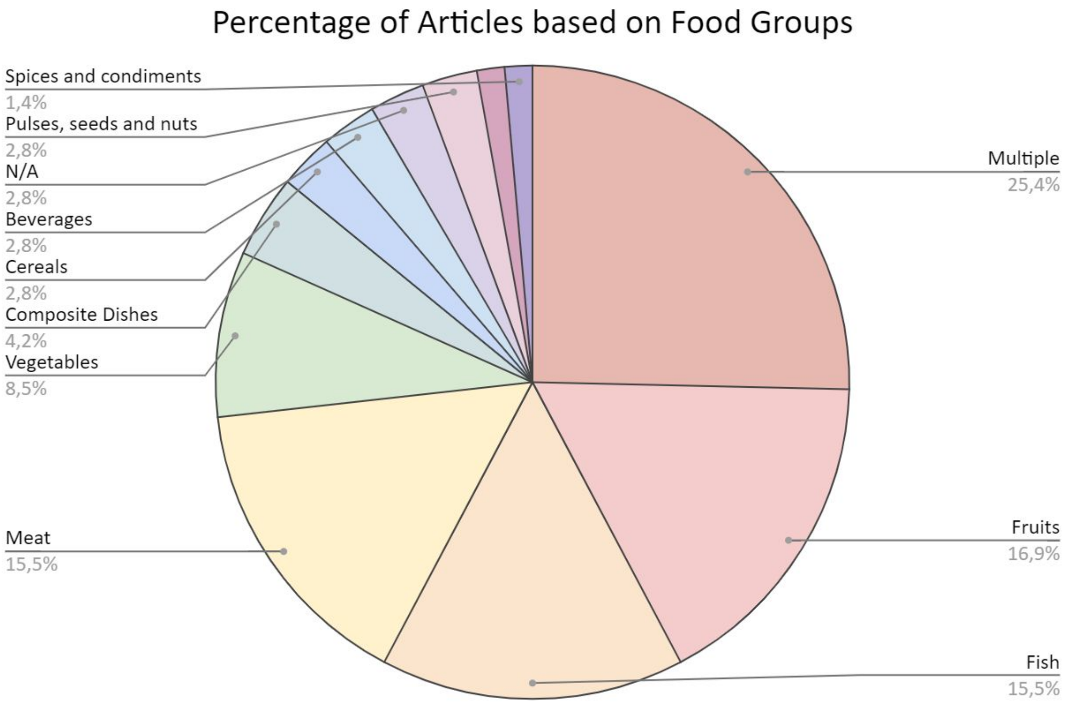

Figure S1-4: Publication share by food group

Figure S1-5 illustrates the percentage of articles based on food supply chain stages. Storage with 39,4% dominates the area of research and transportation follows at 19,7%, highlighting tracking and monitoring during transit. Storage and distribution accounts for 14.1% indicated another important food supply chain stage. Moreover, retail at 7% and manufacturing at 5% follow the other, while multiple stages are focused on 12.7%

of the articles. Also, one article focusing on storage at customer level was found. This variability highlights the potential of using IoT sensors for reducing FLW in all stages of the food supply chain.

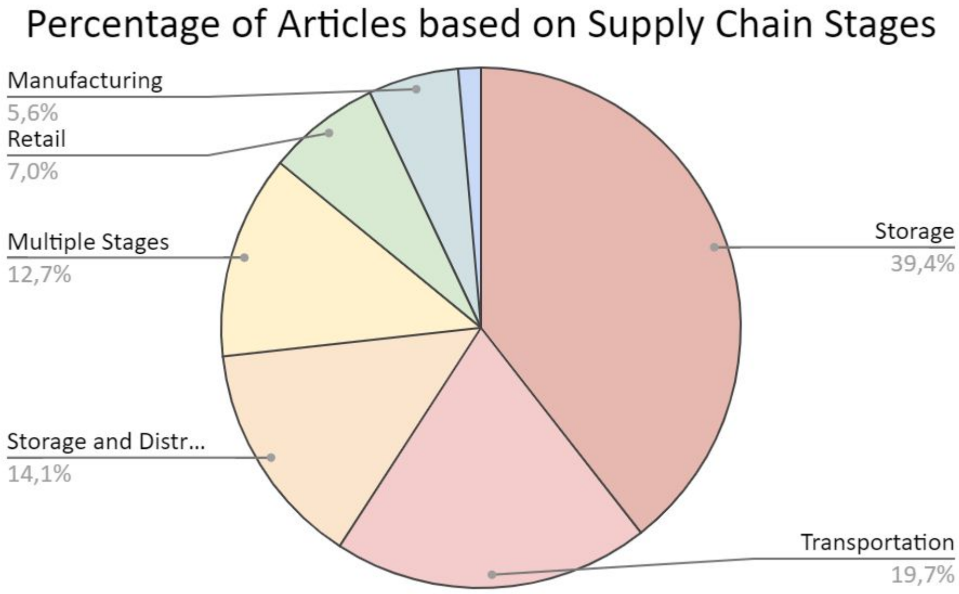

Figure S1-5: Publication share by supply-chain stage

Figure S1-6 illustrates the distribution of articles related to IoT applications in mitigating FLW along food supply chains across various countries revealing the global interest and engagement of researchers. With a total of 19 articles, China takes the lead in the research. This outstanding contribution demonstrates China's initiative in IoT research in the context of FLW prevention and mitigation along food supply chains. The "Multiple Countries" category includes research that exhibits cross-national cooperation. Moreover, five articles each from Italy and Spain show a rising interest in using IoT to reduce FLW along food supply chains in Europe while South Korea represents the growing interest in IoT among Asian countries and four articles are also presented by the United States. Furthermore, various nations, including Brazil, Hong Kong, India, Iran, Portugal, Turkey, have also contributed one to three articles. These contributions demonstrate a widespread interest in examining the possibilities of IoT

technology in the food business, demonstrating the technology's adaptability in tackling a range of food supply chain concerns. Moreover, **Figure S1-6** illustrates the numbers of publications from 2003 to 2022 in different countries on a worldwide basis on the world map. It is clearly seen here that China stands out in the studies.

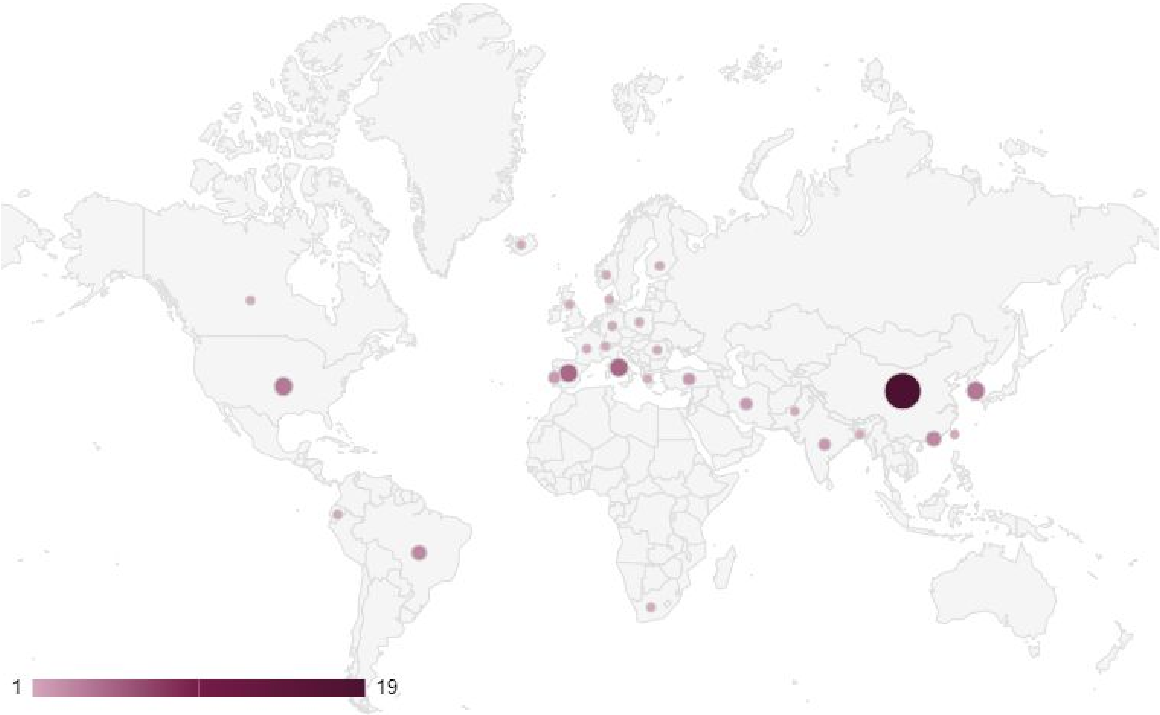

Figure S1-6: Global publication distribution (world map)

Recent progress in food sensing area. This review performed network visualization using keyword co-occurrence analysis in VOSviewer to understand the role of IoT solutions in extending shelf life and reducing FLW along the supply chain (**Figure S1-7**). Analyzing the co-occurrence of keywords in terms of their frequency and relevance provides an intuitive means to discern research trends and identify cutting-edge topics. The study searched full documents (articles and early access) of papers in WoS published from 2003 to 2022 with the keywords "shelf life" OR "food waste*" OR "food loss*" AND "smart packaging" OR “sensor” AND "dynamic" OR “real-time” OR "internet of things" OR "IoT". We then created a term co-occurrence map based on text data and used full counting in this review, showing 69 items (7 clusters) and 960

links. The minimum number of occurrences of a term is set to be 5. Based on the scope of the threshold, the number of most relevant terms is to be selected 84. As summarized in **Table S1-1**, it is evident that Cluster 1 boasts the highest number of keywords, signifying a centralization of identical themes related to IoT solutions for addressing FLW. The main keywords ranked by occurrence or frequency are “supply chain”, “iot”, “food waste”, “freshness”, “fruit”, “degrees c (temperature)”, “packaging”, “thing”, “detection”, “real time” (Table 2: the top effective keywords). Meanwhile, keywords like “iot”, “algorithm”, “sensor”, “smart packaging”, “wireless sensors network”, “radio frequency identification”, “block chain”, “traceability”, continue to underscore the research hotspots within the technologic domain. On the other hand, the review observed more specific keywords, such as “fruit”, “meat”, “production”, “cold storage”, “cold chain”, “online retailer”, “consumer”, “label”, suggesting the emergence of more actual case studies. Most importantly, this review noticed a shift in keywords post-2020 toward increased environmental and social concerns, such as “environmental impact”, “life cycle assesment”, “co2”, “shelf life extention”, “reduction”, “quality loss”, “marketability”, “critical quality parameter”.

Table S1-1: Keyword clusters and counts

Cluster	Term	Selected keywords
1	15	accuracy; algorithm; cold storage; color; detection; e nose; freshness; humidity sensor; label; meat; real time; smart packaging; spoilage; temperature sensor; test
2	14	coating; environmental impact; food loss; food packaging; food spoilage; food waste; life cycle assessment; microbial growth; nano packaging; production; reduction; shelf life extension; use
3	11	architecture; China; co2; decision; honey peach export chain; quality control; relative humidity; traceability system; transparency; wireless sensors network; wsn
4	10	consumer; mathematical model; online retailer; perishable product; quality assurance; radio frequency identification; retailer; rfid; social learning; user

5	9	advantage; cold chain; implementation; internet; iot; major challenge; product challenge; product quality; thing; wireless sensor network
6	5	criterium; fruit; marketability; quality loss; supply chain
7	5	block chain; critical quality parameter; degrees c; experiment; traceability

Table S1-2: Top-30 keywords by occurrence

Ranking	Keyword	Occurrence	Ranking	Keyword	Occurrence
1	supply chain	44	16	decision	14
2	iot	40	17	spoilage	14
3	food waste	36	18	consumer	14
4	freshness	30	19	food loss	13
5	fruit	29	20	label	13
6	degrees c	25	21	meat	12
7	packaging	24	22	experiment	12
8	thing	24	23	relative humidity	12
9	detection	24	24	environmental impact	12
10	real time	23	25	traceability	12
11	wsn	22	26	algorithm	11
12	production	20	27	retailer	11
13	reduction	19	28	architecture	11
14	cold chain	18	39	wireless sensor network	11
15	rfid	15	30	quality control	11

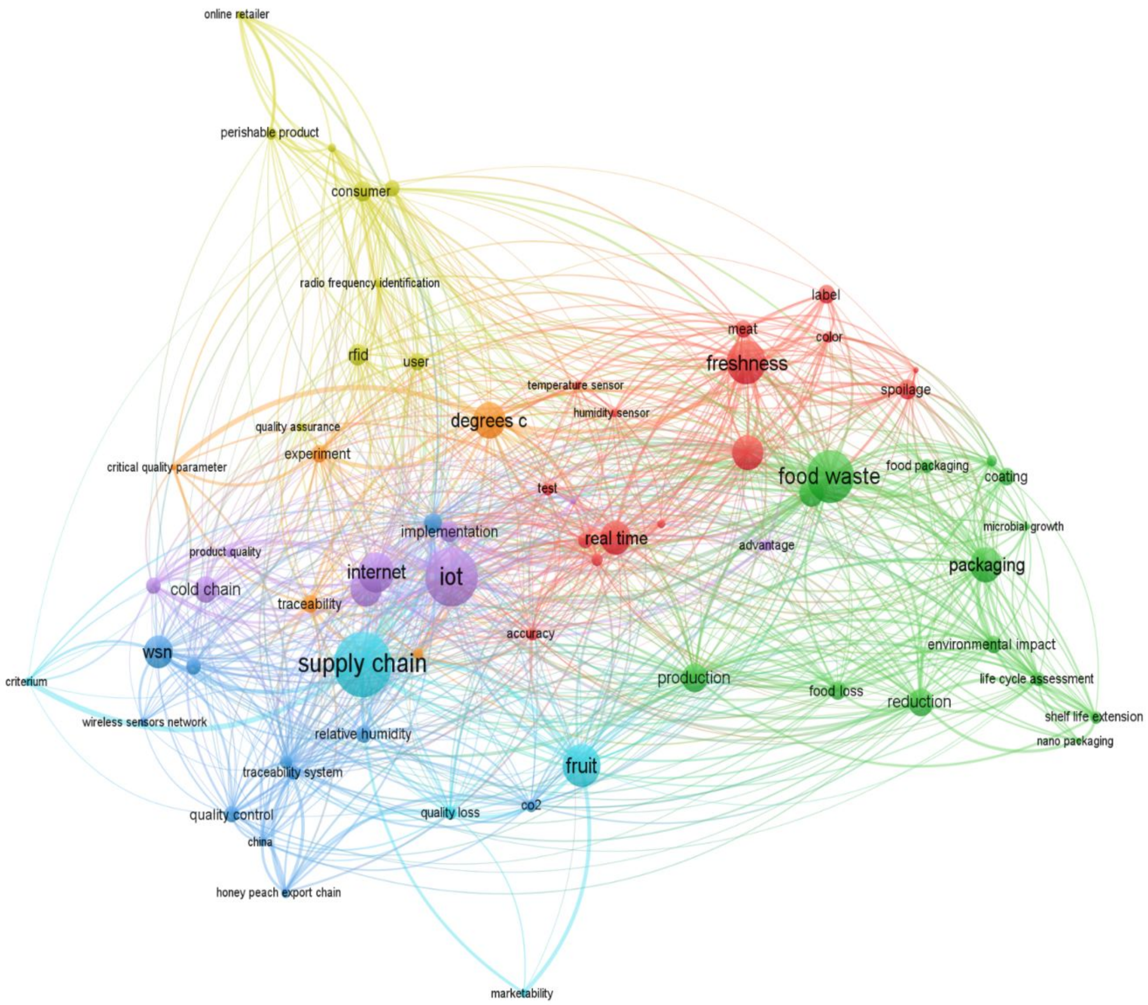

Figure S1-7: Keyword co-occurrence network (VOSviewer)

2 Development of Shelf Life – Food Waste – IoT Sensor Models

2.1 System Definition and Assumption

Figure S1 provides a snapshot of the food chain, highlighting the journey of fresh food as it moves from suppliers to retail stores and ultimately to consumers. Our study specifically focuses on the retail segment of this chain, excluding the initial food production phase and the disposal of unsold or spoiled food. In this data-driven DSL system, food is classified as waste not based on the printed expiry date but on the actual deterioration of food quality below a predefined threshold (**Figure S1a**). Conversely, under the fixed shelf life (*FSL*) system, food items are considered food waste once they surpass their labeled expiry date (**Figure S1b**). This distinction in the criteria for determining the percentage of food waste illustrates the differing operational principles of the DSL and FSL systems. The key processes involved in this system are detailed below:

2.1.1 Supplier Process

In the initial phase of the food chain, following the placement of an order by a retail entity, suppliers expedite the dispatch of fresh food items from their logistical hubs to the respective retail destinations within a predefined time window. This study posits a many-to-many interaction paradigm between suppliers and retailers, characterized by normally distributed variance in food quality. The heterogeneity in the quality of food consignments is an inherent attribute, resulting from the complex dynamics of logistic operations prior to final delivery to retail outlets. Additionally, disparities in the quality of individual food batches arise from differences in thermal sensitivities, further accentuating quality variance.

In this study, storage temperatures for vegetables, fruits, and dairy products were maintained between 0 and 8°C. Conversely, meat and fish & seafood products were

stored between -20°C and -15°C. Ensuring these optimal temperature conditions is crucial for maintaining the quality-centered DSL management system within temperature-sensitive food chains. Shelf life data at specific temperatures across different food products were collected. While temperature fluctuations are predominant determinants of shelf life, the initial quality of the food remains a pivotal factor. Generally, the perishability function of a food product can be scaled based on the acceptable quality levels of certain measurable parameters. It is assumed that the perishability function falls within the real interval [0, 1], where a value of 1 indicates that the product has retained its original quality without degradation, and a value of 0 signifies that the product holds no value or utility. For our analysis, we assumed that the perishability function of food products lies within the ranges [0.9, 0.1], [0.7, 0.2], and [0.5, 0.3] to estimate their respective shelf lives and food waste under consistent storage temperatures. These ranges represent the most to the least efficient scenarios within the food chain.

2.1.2 Retail Process

In the context of contemporary retail operations, activities related to food delivery include the reception of purchased items, sales facilitation, formulation of order policies, and inventory management. Before food batches are placed on retail displays, a mandatory quality assessment is conducted. This study hypothesizes that food batches with an initial quality below 30% are deemed unacceptable and thus rejected. Temperature variability, influenced by factors such as time and spatial characteristics like shelf location and design, is notable even among food items displayed for sale. Generally, the state of fresh food within retail environments can be categorized into three distinct phases: displayed, sold, and wasted.

The deployment of a Wireless Sensor Network (*WSN*) system enables autonomous tracking of the residual quality or shelf life of all fresh food items by monitoring their temperature and humidity and using a predictive model for shelf life estimation. In this

data-driven DSL system, the predicted shelf life of food items can be dynamically adjusted, potentially preventing premature disposal. Food items falling below a specified quality threshold, thereby becoming unsalable, are discarded. Here, 'perishing' refers to the degradation of food quality to levels below the set threshold for salability. This analysis sets the replenishment cycles for fresh food from suppliers to retailers at one day, as delineated by Salinas Segura and Thiesse (2017). [8]

2.1.3 Consumer Process

The operational dynamics of the system are primarily driven by consumer demand for fresh food products, as depicted in **Figure S1**. For analytical purposes, each delivery of fresh food is disaggregated into individually identifiable units. The model assumes a uniform distribution for the intervals between successive consumer arrivals at retail outlets. Additionally, it is hypothesized that the daily influx of consumers follows a truncated normal distribution.

In terms of consumer behavior, it is assumed that consumers exhibit rational decision-making processes. Under the data-driven DSL system (**Figure S1a**), consumers are expected to preferentially select the freshest available products. Conversely, within the traditional FSL framework (**Figure S1b**), it is assumed that consumers will gravitate towards products with the longest indicated shelf life. The occurrence of an unstocked shelf is interpreted within the model as an indicator of stockout conditions.

S2.1.4 Ordering Policy

Retailers routinely check their inventory at predetermined intervals and are permitted to place orders daily. The ordering process involves calculating the difference between current stock levels and a pre-set replenishment target. This study assumes a consistent replenishment cycle, accompanied by a specific service level, to assess the impact of the ordering policy in both data-driven DSL and traditional FSL frameworks. In this

context, the ‘service level’ refers to the likelihood of avoiding stockouts and serves as a measure of the reliability in meeting consumer demand. It is inversely related to the out-of-stock rate. Typically, a higher service level necessitates larger order quantities. This approach effectively minimizes stockouts without altering overall consumption levels. However, it also tends to result in the accumulation of more stock on delivery days, particularly for perishable items with short shelf lives, potentially leading to increased food waste.

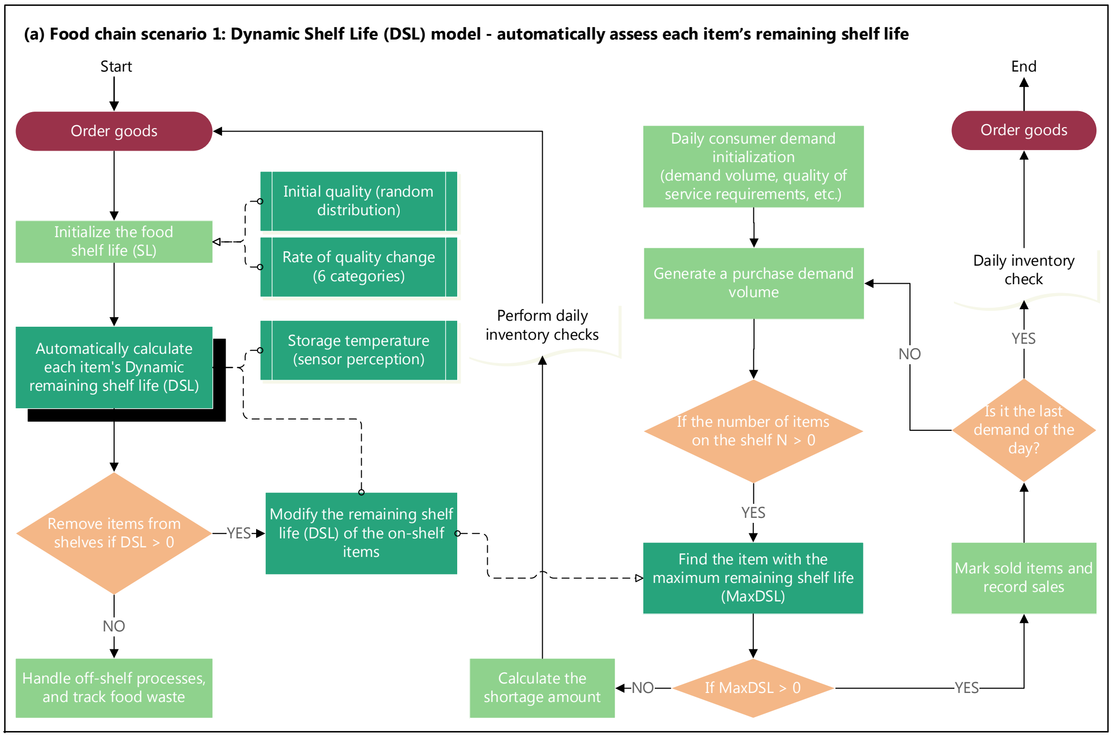

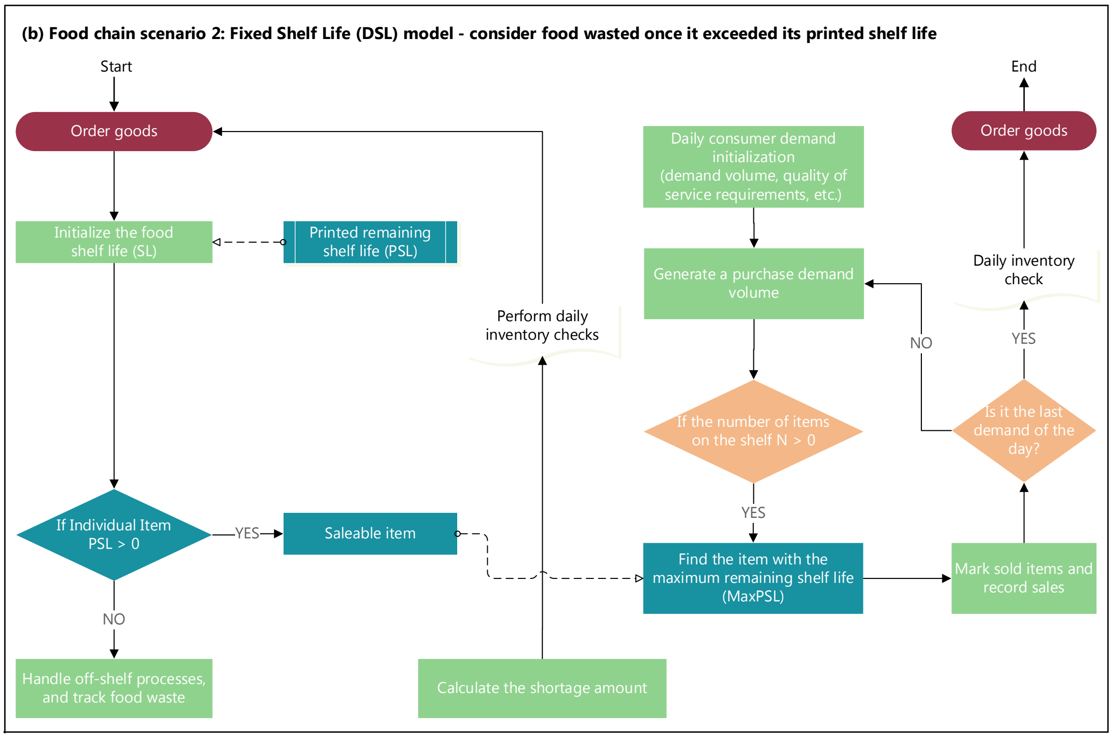

Figure S2-1: FSL vs. DSL system framework for temperature-sensitive foods

S2.1.5 Monte Carlo Simulation

We applied a Monte Carlo simulation to assess the dynamic changes in shelf life and the associated food waste potentials of perishable food products. The simulation involved the following key steps and considerations:

- **Randomized Parameters**. The simulation employed a randomized approach to capture variations in storage temperature, perishability, and storage duration, reflecting real-world fluctuations occurring throughout the cold chain process. For each iteration, storage temperatures were randomized within predetermined ranges to simulate potential variations during the shelf storage phase. Specifically, we defined three temperature zones: low (0 to 8°C), moderate (12 to 18°C), and high (20 to 28°C), with individual storage temperatures following a homogenous random distribution within these ranges. For storage duration, we assumed daily demand at retail stores to follow a truncated normal distribution with a mean (μ) of 50 units and a standard deviation (σ) of 10, truncated at a value of 30 units. Purchasing demand

was distributed evenly throughout the day, with each instance of demand treated as a decision point. During each decision point, the storage time for all individual food products was incremented by an interval until they were purchased. Considering the actual operation of retail stores, each operational day was defined as 0.5 days (12 hours). At the end of each operational period, inventory was reviewed, and products with a remaining shelf life of less than 0.5 days were removed from shelves, while the storage time for all other products was extended by 0.5 days.

- **Temperature and Quality Rate Calculations**. The Monte Carlo simulation utilized three reference temperatures, and corresponding quality deterioration rates were calculated based on these temperatures. The quality deterioration rate at different reference temperatures was modeled using the temperature coefficient method (see the corresponding formula in subsection 2.3). This allowed for calculating the spoilage rate based on temperature deviations from a baseline value. The random temperature values were used to adjust the quality deterioration rate accordingly.
- **Shelf-Life Estimation**. The initial shelf life of each product was determined by a truncated normal distribution [0.3, 0.95], representing the inherent variability in product quality. For each product, the remaining shelf life was recalculated for each time step based on the randomized storage temperatures and the quality change rates. If the remaining shelf life of a product fell below a certain threshold (determined by a safety margin), it was considered expired and marked for removal.
- **Consumer Demand Simulation**. The simulation also included a model for purchase intervals that were distributed evenly throughout the day, and the simulation calculated how much of the perishable inventory was purchased versus how much expired without being sold.
- **Iteration Process**. The Monte Carlo simulation was iterated over multiple cycles (100 for this study) to capture a range of possible outcomes for food quality and shelf life. Each iteration tracked the number of safe products sold, expired products, and unsold but still safe products. The outputs from each iteration were then aggregated

to determine key metrics such as average remaining shelf life, food waste rates, and demand satisfaction rates.

- **Outcome Analysis**. The results of the Monte Carlo simulation were analyzed to evaluate the shelf life associated with perishable food loss, as well as to determine the effectiveness of cold chain management in extending the shelf life of different food categories. Statistical measures such as standard deviation were used to represent the variability across different iterations, providing a comprehensive understanding of how uncertainties in cold chain management impact food shelf life and food waste potential.

2.2 Development of Shelf Life – Food Waste Management Models

Food spoilage arises from a complex interplay of biological, physical, and chemical factors [9], often beginning with imperceptible microbial growth that eventually leads to noticeable quality deterioration. [10] Numerous models predicting the shelf life of fresh foods rely on the Arrhenius law. [11] In this study, we employ a kinetic approach to quantify quality degradation as a function of temperature, thereby linking shelf-life changes to temperature-dependent food losses. The process is described by Eq. (S2-1):

$$\frac{dQ}{dt} = kQ^n \qquad \text{(S2-1)}$$

where Q is the food quality, k is the reaction rate, n is the reaction order, and t is time.

The reaction rate k commonly follows an Arrhenius-type relationship with temperature:

$$k(T) = k_0 e^{-\frac{E_a}{RT}} \qquad \text{(S2-2)}$$

where $k(T)$ is the reaction rate at temperature T (in Kelvin), k_0 is a known reaction rate at a reference temperature, E_a is the activation energy (food-specific), and R is the universal gas constant (8.314 J·mol^{-1}·K^{-1}).

To simplify this formulation, many studies replace the explicit activation energy with the Q_{10} factor, defined as the ratio of reaction rates at $T + 10$ °C and T °C. [11] Empirical findings suggest Q_{10} values ranging from 2 to 4 for 0–10 °C. [12,13] Here, we select $Q_{10} = 3$ to balance model complexity and uncertainty in fresh food quality changes. [14] Although predictive accuracy may vary, the model's error remains acceptable over small temperature interval [15]:

$$Q_{10} = \frac{k(T+10)}{k(T)} \qquad \text{(S2-3)}$$

Assuming a zero-order reaction ($n = 0$), we approximate quality loss as linear with respect to time. For a temperature increase $\Delta T \in [0,10]$ °C, the relative change in reaction rate is:

$$Q_{\Delta T} = \frac{k(T+\Delta T)-k(T)}{k(T)} = (Q_{10} - 1) \cdot \frac{\Delta T}{10} \qquad \text{(S2-4)}$$

Accordingly, the new reaction rate at $T + \Delta T$ is:

$$k(T + \Delta T) = \left[1 + (Q_{10} - 1) \cdot \frac{\Delta T}{10}\right] \times k(T) \qquad \text{(S2-5)}$$

From this relationship, the remaining shelf life (SL) of fresh food at temperature $T + \Delta T$ after time t is:

$$SL = SL_0 - \left[1 + (Q_{10} - 1) \cdot \frac{\Delta T}{10}\right] \times k(T) \times t \qquad \text{(S2-6)}$$

where SL_0 is the initial shelf life prior to entering the retail stage.

The static shelf life of perishable foods is determined based on the maximum recommended storage temperature (e.g., -15°C for frozen fish or +8°C for fresh milk).[16] However, due to temperature fluctuations throughout the cold chain, the dynamic shelf life (DSL) often diverges from the fixed SL. Under conditions where temperatures remain below the maximum recommended storage temperature, the actual shelf life of

perishable foods is extended. This discrepancy means that some products deemed expired based on static SL information may still be suitable for consumption based on their DSL.

Assuming a constant daily food sales volume, denoted as D, the food waste generated due to inaccuracies in static SL systems, which can be mitigated by adopting a DSL system, is given by Eq. (S2-7):

$$FW = D \times (SL_{dynamic} - SL_{fixed}) \qquad \text{(S2-7)}$$

The corresponding food waste rate (FWR) is calculated in Eq. (S2-8):

$$FWR = \frac{FW}{D \times SL_{fixed}} = 1 - \frac{SL_{dynamic} - SL_{fixed}}{SL_{fixed}} \qquad \text{(S2-8)}$$

Here, FWR reflects the change in food loss rate caused by the DSL management system, which may be positive or negative. A negative FWR indicates a reduction in food loss due to the implementation of a DSL system.

By integrating temperature-sensitive DSL systems, stakeholders can dynamically adjust shelf life estimates, minimize food waste, and optimize supply chain operations. This approach underscores the value of real-time temperature monitoring in enhancing sustainability and reducing losses in perishable food supply chains.

2.3 Data Collection and Quality

The primary objective of this research was to quantitatively assess the impact of the Dynamic Shelf Life (DSL) system on food waste reduction in comparison to traditional Fixed Shelf Life (FSL) for fresh produce. The scope encompasses various food categories, including fruits, vegetables, dairy, and frozen meat and aquatic products, within the retail sector. This phase involved collecting and analyzing data on the shelf life and waste rates of selected food categories. The study utilized a self-developed

computer-simulated platform for analyzing variations in food waste rates (The code is provided upon reasonable request). The data for this study is contextualized within the geographical confines of Mainland China and is specific to the year 2020. Representative data on the shelf life of these food categories were sourced from *'Food Cold Chain Logistics - Temperature Control Requirements for Storage, Transport and Distribution*'. [17] Data across other food stages, incl. production, postharvest handling, storage, transport, distribution center (DC) and retail are derived from Lu et al. (2022) and Wang et al. (2023). [18,19]

In adherence to the principles of mass balance, this study rigorously documented the total input of fresh produce and the corresponding output, defined as the discrepancy in produce classified as waste before and after the implementation of the DSL system. By-products in the food supply chain were not considered. This approach ensured a balanced account of material inputs and outputs, thereby facilitating an accurate evaluation of the DSL system's impact on food waste.

This research established a critical threshold, designating fresh produce with less than one day of remaining shelf life as waste. This threshold played a pivotal role in our analysis, enabling us to accurately calculate the food waste rate, a key metric in evaluating the DSL system's impact. The DSL system's effectiveness was evaluated by comparing the shelf life and waste rates between the DSL system and traditional expiration date methods. The comparison was contextualized across different stages of the food supply chain, namely production, postharvest handling, packaging, storage, transport, distribution, and retail. The DSL system, leveraging sensor-based technologies, predicts the remaining shelf life of products, allowing for timely removal from shelves to minimize waste. The statistical analysis of two system performance in refrigerated temperature ranges is presented in **Table S2-8**.

The findings indicated a potential reduction in food waste through the implementation of the DSL system. This was quantified by aggregating the waste rate for each food

type at each stage before reaching the consumer. Additionally, the study explored the implications of waste reduction in the retail sector and its potential to decrease waste in other sectors. The equation below was used to convert the avoided retail waste into savings in other areas of the supply chain.

The following **Tables S2-1 to S2-7** offer a comprehensive database of various fresh produces, encompassing 58 types of perishable foods across a spectrum of temperature conditions and diverse quality characteristics. The detailed Tables correlate shelf life with relative temperature conditions throughout the supply chain.

Table S2-1: List of 58 selected fresh foods

No.	Fresh food	Retail shelf/ ℃	Recommended storage time range (d)
1	Longan, litchi	4	21
2	Mango	14	18
3	Papaya	10	8.5
4	Cherimoya	17.5	25
5	Pineapple	10.5	17.5
6	Banana	13.5	17.5
7	Strawberry	1.5	10.5
8	Grape	1.5	66.5
9	Guava	7.5	17.5
10	Carambola	7.5	24.5
11	Citrus	6	45
12	Pomelo	7.5	75
13	Lemon	11.5	105
14	Grapefruit	12.5	49
15	Apple, pear	2	120
16	Jujube	6	180
17	Cherry	1.5	17.5
18	Plum	1.5	24.5
19	Peach	1.5	24.5
20	Watermelon/ melon	8.5	17.5
21	Radish	1.5	24.5
22	Carrot	1	90
23	Asparagus	1	17.5
24	Potatoes	3.5	240

No.	Fresh food	Retail shelf/ ℃	Recommended storage time range (d)
25	Onions	1	180
26	Cauliflower	1	17.5
27	Chinese chive flowers	1	14
28	Day lily	1	6.5
29	Cabbage [1]	1	85
30	Lettuce	1	22.5
31	celery	1	24.5
32	Cabbage [2]	1	55
33	Spinach	1	22.5
34	Rapeseed	1	25.5
35	Chinese Kale	1	29.5
36	Ripe green tomato	15	14
37	First ripe tomato	8.5	5.5
38	Ripe tomato	2.5	14
39	Sweet corn	1	10
40	Pumpkin	12.5	75
41	Cucumber	11.5	12
42	Peas	1	19
43	Edamame	1	24.5
44	Flammulina velutipes	1	22.5
45	Garlic	2.5	180
46	Onions, garlic sprout	2.5	22
47	Chili	8	27.5
48	Tender ginger	13	18
49	Ginger	13	120
50	Chilled aquatic products	4	2
51	Fresh animal meat	2	3
52	Chilled livestock and poultry	2	3
53	Chilled processing seafood pickle products	2	45
54	Pasteurized milk	4	10 [a]
55	Fresh eggs	4	45 [a]
56	Lactic acid bacteria drink	6 [a]	30 [a]
57	Flavor yogurt	4 [a]	24 [a]
58	Fresh milk	6 [a]	21 [a]

Note: *[a] refers to the storage temperature and shelf life marked by the manufacturer on the fresh food product based on the market survey in Guangzhou supermarket in China.*

Table S2-2: Fruit cold-chain temperature requirements

Category	Sub-category	Pre-cooling / ℃	Storage / ℃	Storage humidity / %	Transportation		Retail shelf/ ℃	Recommended storage time range
					Medium and long-distance/ ℃	Short-range delivery (< 5h)/ ℃		
Large berries	Longan/ litchi	3 – 5	3 – 5	90 – 95	3 – 5	5 – 12	3 – 5	2 – 4 weeks
	Mango	13	13 – 15	85 – 90	13 – 15		13 – 15	2 – 3 weeks
	Papaya	7 – 10	7 – 13	85 – 90	7 – 13		7 – 13	7 – 10 days
	Cherimoya	7 – 10	15~20	85 – 90	15 – 20		15 – 20	3 – 4 weeks
	Pineapple	10 – 15	8 – 13	85 – 90	8 – 13		8 – 13	2 – 3 weeks
	Banana	13 – 14	12 – 15	80 – 90	12 – 15		12 – 15	1 – 4 weeks
Small berries	Strawberry	-1 – 0	0 – 3	90 – 95	0 – 3	5 – 12	0 – 3	1 – 2 weeks
	Grapes	-1 – 0	0 – 3	90 – 95	0 – 3		0 – 3	7 – 12 weeks
	Guava	5 – 10	5 – 10	90 – 95	5 – 10		5 – 10	2 – 3 weeks
	Carambola	5 – 10	5 – 10	85 – 90	5 – 10		5 – 10	3 – 4 weeks
Citrus	Citrus	4 – 8	4 – 8	85 – 95	4 – 8	5 – 12	4 – 8	1 – 2 months
	Pomelo	5 – 10	5 – 10	85 – 90	5 – 10		5 – 10	2 – 3 months
	Lemon	11 – 13	10 – 13	85 – 90	10 – 3		10 – 13	1 – 6 months
	Grapefruit	10 – 15	10 – 15	85 – 90	10 – 15		10 – 15	6 – 8 weeks
Pomaceous fruits	Apple/ pear	0 – 1	0 – 4	90 – 95	0 – 4	5 – 12	0 – 4	1 – 6 months
	Jujube	0 – 2	5 – 7	90 – 95	5 – 7		5 – 7	6 – 12 months
	Cherry	0 – 2	0 – 3	90 – 95	0 – 3		0 – 3	2 – 3 weeks
	Plum	0	0 – 3	90 – 95	0 – 3		0 – 3	2 – 5 weeks
	Peach	0	0 – 3	90 – 95	0 – 3		0 – 3	2 – 4 weeks
Melons	Watermelon/ melon	10 – 15	7 – 10	85 – 90	7 – 10	5 –12	7 –10	2 – 3 weeks

Table S2-3: Vegetable cold-chain temperature requirements

Category	Sub-category	Pre-cooling / ℃	Storage / ℃	Storage humidity /%	Transportation: Medium and long-distance/ ℃	Transportation: Short-range delivery (< 5h)/ ℃	Retail shelves/ ℃	Recommended storage time range
**Root vegetables**	Radish	0 – 1	0 – 3	90 – 95	0 – 3	5 – 12	0 – 3	3 – 4 weeks
	Carrot	0 – 1	0 – 2	95 – 100	0 – 2		0 – 2	3 – 5 months
	Asparagus	~ 7	0 – 2	95 – 100	0 – 2		0 – 2	2 – 3 weeks
	Burdock	0 – 1	0 – 2	95 – 100	0 – 2		0 – 2	NA
	Potatoes	0 – 1	2 – 5	90 – 95	2 – 5		2 – 5	8 – 9 months
	Onions	0 – 1	0 – 2	65 – 70	0 – 2		0 – 2	6 – 8 months
**Flower vegetables**	Cauliflower	5	0 – 2	≦ 95	0 – 2	5 – 12	0 – 2	3 – 4 weeks
	Chinese chive flowers	0 – 1	0 – 2	≦ 95	0 – 2		0 – 2	12 – 16 days
	Day lily	0 – 1	0 – 2	≦ 95	0 – 2		0 – 2	6 – 7 days
	Cabbage	0 – 1	0 – 2	95~98	0 – 2		0 – 2	80 – 90 days
	Lettuce	0 – 1	0 – 2	95 – 98	0 – 2		0 – 2	17 – 28 days
**Leafy vegetables**	Celery	0 – 1	0 – 2	≦ 98	0 – 2	5 – 12	0 – 2	3 – 4 weeks
	Cabbage	0 – 1	0 – 2	≦ 98	0 – 2		0 – 2	40 – 70 days
	Garden chrysanthemum	0 – 1	0 – 2	≦ 98	0 – 2		0 – 2	NA
	Spinach	0 – 1	0 – 2	≦ 95	0 – 2		0 – 2	20 – 25 days
	Rapeseed	0 – 1	0 – 2	≦ 95	0 – 2		0 – 2	21 – 30 days
	Chinese Kale	0 – 1	0 – 2	≦ 95	0 – 2		0 – 2	25 – 34 days
**Fruit vegetables**	Ripe green tomato	9 – 10	14 – 16	90 – 95	14 – 16	5 – 12	14 – 16	1 – 3 weeks
	First ripe tomato	9 – 10	7 – 10	90 – 95	7 – 10		7 – 10	4 – 7 days
	Ripe tomato	9 – 10	0 – 5	90 – 95	0 – 5	\	0 – 5	1 – 4 weeks
	Sweet corn	0	0~2	95 – 98	0 – 2	\	0 – 2	8 – 12 days
	Pumpkin	7 – 10	10 – 15	50 – 70	10 – 15	5 – 12	10 – 15	2 – 3 months
	Cucumber	7 – 10	10 – 13	90 – 95	10 – 13	\	10 – 13	10 – 14 days
	Peas	9 – 10	0 – 2	95 – 98	0 – 2	5 – 12	0 – 2	18 – 20 days
	Edamame	9 – 10	0 – 2	95 – 98	0 – 2	\	0 – 2	21 – 28 days
	Twin mushroom (mushroom)/ flammulina velutipes	0	0 – 2	≦ 95	0 – 2	5 – 12	0 – 2	45 days
	Garlic (naked garlic)	0	0 – 5	65 – 70	0 – 5	5 – 12	0 – 5	6 – 7 months
	Onions/ garlic sprout	0	0 – 5	95 – 100	0 – 5	\	0 – 5	18 – 26 days
	Chili	7 – 10	7 – 13	90 – 95	7 – 13	\	7 – 13	25 – 30 days
	Tender ginger	13	13	90 – 95	13	\	13	16 – 20 days
	Ginger	13	13	65 – 80	13	\	13	4 – 5 months

Table S2-4: Aquatic-product temperature requirements

Category	Storage/ ℃	Transportation		Retail shelves / ℃	Processing zone / ℃	Recommended storage time range
		Medium and long-distance/ ℃	Medium and long-distance/ ℃			
Frozen aquatic products	≧ –18	≧ –18	≧ –12	≧ –12	≧ 10	6 months
Chilled aquatic products	≧ 4	≧ 4	≧ 4	≧ 4	12 – 15	2 days
Frozen aquatic products	≧ –18	≧ –18	≧ –12	≧ –12	≧ 10	6 months
Chilled aquatic products	≧ 4	≧ 4	≧ 4	≧ 4	Cold water ≦ 10 Warm water ≦ 15	7 days
Chilled processing seafood pickle products	≧ 4	≧ 4	≧ 4	≧ 4	12 – 15	15 days
Ultra-low temperature frozen aquatic products	≧ –50	≧ –50	≧ –30	≧ –30	NA	6 months

Table S2-5: Livestock and poultry meat temperature requirements

Category	Storage/ ℃	Transportation		Retail shelves/ ℃	Processing zone / ℃	Recommended storage time range
		Medium and long-distance/ ℃	Medium and long-distance/ ℃			
Frozen livestock and poultry	≧ –18	≧ –18	≧ –12	≧ –12	≧ 0	6 months
Chilled poultry/ fresh poultry	≧ 4	≧ 4	≧ 4	≧ 4	15	3 days
Frozen processing pickled poultry meat	≧ –18	≧ –18	≧ –12	≧ –12	4	6 months
Cooling processing pickled livestock and poultry meat	≧ 4	≧ 4	≧ 4	≧ 4	4	30 – 60 days

Table S-6: Other fresh-food temperature requirements

Category	Storage/ ℃	Transportation		Retail shelves/ ℃
		Medium and long-distance/ ℃	Medium and long-distance/ ℃	

**Quick-frozen prepared food**	≧ –18	≧ –18	≧ –12	≧ –12
**Chilled prepared food**	≧ 5	≧ 5	≧ 5	≧ 5
**Frozen egg**	≧ –18	≧ –18	≧ –12	≧ –12
**Chilled egg**	≧ 4	≧ 4	≧ 4	≧ 4
**Liquid milk**	≧ 5	≧ 5	≧ 5	≧ 5
**Chilled baked food**	≧ 5	≧ 5	≧ 5	≧ 5
**Chocolate**	15 – 20	15 – 20	15 – 20	15 – 20
**Cream**	≧ –18	≧ –18	≧ –12	≧ –12
**Ice cream**	–25	≧ –18	≧ –18	≧ –18
**Soy products**	≧ 5	≧ 5	≧ 5	≧ 5
**Fruit juice**	≧ 5	≧ 5	≧ 5	≧ 5

Table S2-7: Retail shelf temperatures for fresh foods

**Food category**	**Size of store**	**Number of samples**	**Recommended shelves temperature**	**Measured food temperature**				**Reference**
				Min	Max	Average	Distribution	
**Poultry**	Small	7	0 – 4	3.0	5.1	4.1	NA	20
	Medium	26		2.0	7.0	4.5	NA	20
	Large	76		2.0	2.0	2.0	NA	20
**Butter**	Small	119	2 – 6	5.0	14.0	9.5	NA	20
	Medium	46		8.0	16.0	12.0	NA	20
	Large	130		3.5	6.0	4.7	NA	20
**Yogurt**	Small	46	2 – 6	2.8	10.5	6.7	NA	20
	Medium	113		2.5	13.0	7.7	NA	20
	Large	199		5.0	9.0	7.0	NA	20
**Eggs**	Small	53	≥ 4	8.0	17.0	12.5	NA	20
	Medium	96		6.0	8.5	7.2	NA	20
	Large	96		3.9	9.3	6.6	NA	20
**Milk**	Large	50	2 – 6	4.5	11.2	7.8	NA	b
**Frankfurters**	Small	31	4	4.9	10.5	7.7	NA	20
	Medium	79		5.0	8.0	6.5	NA	20

Food category	Size of store	Number of samples	Recommended shelves temperature	Measured food temperature				Reference
				Min	Max	Average	Distribution	
	Large	56		–1.1	8.5	3.1	NA	20
Cheese	Small	23	1 – 3	4.0	7.5	5.8	NA	20
	Medium	43		4.0	8.0	6.0	NA	20
	Large	74		5.0	5.0	5.0	NA	20
Ice cream	Small	29	< –18	-23.0	-10.0	-16.5	NA	20
	Medium	49		-18.5	-13.0	-15.7	NA	20
	Large	79		-14.0	-12.5	-13.3	NA	20
Prepared meat and meat products	NA	307	0 – 4		10.2	3.2	Normal	21
Dairy product (yogurt)	NA	307			9.8	4.2	Normal	21
'Golden Crown' apple (bagged)	NA	NA	4.0	5.8	6.7	6.3	NA	22
'Mackintosh Red' Apple (in bulk)	NA	NA	4.0	6.2	8.3	7.0	NA	22
Banana	NA	NA	13.0 – 15.0	4.6	5.1	4.8	NA	22
BlackBerry	NA	NA	-0.5 – 0.0	6.7	7.9	7.1	NA	22
Blueberry	NA	NA	-0.5 – 0.0	6.6	8.4	7.3	NA	22
Raspberry	NA	NA	-0.5 – 0.0	6.8	7.5	7.2	NA	22
Strawberry	NA	NA	0.0	4.2	8.8	5.7	NA	22
Cucumber	NA	NA	10.0 – 12.0	4.3	8	5.8	NA	22
Grape, red	NA	NA	-0.5 – 0.0	6.1	7.6	6.8	NA	22
Grape, white	NA	NA	-0.5 – 0.0	3.7	6.9	5.7	NA	22

Food category	Size of store	Number of samples	Recommended shelves temperature	Measured food temperature				Reference
				Min	Max	Average	Distribution	
**Fresh-cut vegetables**	NA	NA	1.0 – 3.0	6.8	8.5	7.8	NA	22
**Mango**	NA	NA	13.0	4.4	11.6	8.1	NA	22
**Peach**	NA	NA	-0.5 – 0.0	4.1	6.7	5.3	NA	22
**Pepper, green**	NA	NA	7.0 – 10.0	5.1	7.2	6.0	NA	22
**Pineapple**	NA	NA	7.0 – 13.0	4.6	5.1	4.8	NA	22
**Salad bags (vegetables)**	NA	NA	1.0 – 3.0	6.8	8.1	7.5	NA	22
**Tomato, grape**	NA	NA	8.0 – 10.0	9.9	11.3	10.7	NA	22
**Tomato, cluster**	NA	NA	8.0 – 10.0	8.2	10.5	9.7	NA	22

Note: [b] *refers to the storage temperature and shelf life marked by the manufacturer on the fresh food product based on the market survey in Guangzhou supermarket in China. And NA is not applicable or not available.*

Table S2-8: Statistical performance in refrigerated ranges

Food category	No.	Initial quality of food	Stored temperature (℃)	Remaining shelf life from FSL		Remaining shelf life from DSL	
				Average value	RMSD	Average value	RMSD
Vegetable	N1.1	[0.9,0.1]	[0, 8]	17.42	0.43	17.53	0.31
	N1.2	[0.7,0.2]	[0, 8]	13.22	0.38	13.25	0.36
	N1.3	[0.5,0.3]	[0, 8]	10.96	0.33	10.79	0.43
Fruit	N2.1	[0.9,0.1]	[0, 8]	16.60	0.27	16.55	0.19
	N2.2	[0.7,0.2]	[0, 8]	12.58	0.37	12.62	0.36
	N2.3	[0.5,0.3]	[0, 8]	10.21	0.41	10.41	0.38
Meat	N3.1	[0.9,0.1]	[-20, -15]	80.83	0.98	80.85	0.92
	N3.2	[0.7,0.2]	[-20, -15]	60.79	1.19	60.11	1.18
	N3.3	[0.5,0.3]	[-20, -15]	49.54	1.13	49.28	0.66
Aquatic Products	N4.1	[0.9,0.1]	[-20, -15]	80.83	0.98	80.85	1.34
	N4.2	[0.7,0.2]	[-20, -15]	58.38	1.63	60.11	1.48
	N4.3	·	[-20, -15]	49.54	1.13	49.28	0.66
Dairy	N5.1	[0.9,0.1]	[0, 8]	5.93	0.13	5.96	0.12

	N5.2	[0.7,0.2]	[0, 8]	4.34	0.13	4.58	0.15
	N5.3	[0.5,0.3]	[0, 8]	3.50	0.14	3.90	0.11
Eggs	N6.1	[0.9,0.1]	[0, 8]	16.77	0.22	16.94	0.21
	N6.2	[0.7,0.2]	[0, 8]	12.86	0.41	12.88	0.40
	N6.3	[0.5,0.3]	[0, 8]	10.52	0.38	10.66	0.36

Note: The FSL represents the Fixed Shelf Life (FSL) for various food categories in the absence of any interventions, while DSL refers to the Dynamic Shelf Life of food categories with specific interventions. Remaining shelf life in day (unit). The "RMSE" represents Root Mean Square Deviation.

Based on the above results, the amount of waste reduction from this sensor-based DSL system across food chains was estimated by aggregating the waste rate for each food type at each stage i before consumer (i.e., food production, postharvest handling & packaging, storage, transport & distribution, and retail). The potential waste avoided in the retail sector could be converted into waste saved in other sectors using Eq. S2-9. The corresponding data on avoided food waste rate along the FSC based on the sensor-based DSL system is presented in **Table S2-9**.

$$FWR^{i}_{Convert} = FWR_i \times (1 - \sum_{i=1}^{5} FWR_i) \quad \text{(S2-9)}$$

Table S2-9: Avoided food waste rate along the FSC

Category	Production, %	Post-harvest handling, %	Storage, %	Transportation& Distribution, %	Retail *, %
Vegetable	5.95	6.4	4.0	4.1	6.45 ±1.30
Fruit	5.95	6.4	4	4.1	5.54 ±1.22
Meat	1.33	1.22	1.44	2.43	4.31±0.56
Aquatic	2	3.33	0.79	4.08	4.14±0.54
Milk	0.3	0.6	0.77	2.89	3.43±1.78
Eggs	0.66	0.41	0.65	2.35	6.53±1.63

*Notes *: Data on food waste rate in the retail segment as calculated referring to.* [23] *Data across other food segments are derived from literature studies.* [24,25]

3 Life Cycle Inventory Analysis of Food Supply Chains

3.1 Production Stage

The climate impact of agricultural activities was assessed using the corresponding yields multiplied by the cradle-to-farm emission factor method (See Eq. S3-1). Emission factors for agricultural activities of vegetables, fruit, meats, aquatic products, dairy, eggs were obtained from peer-reviewed scientific literature, and these studies provide comprehensive regional GHG emission values based on food production processes, from cultivation to the farm gate, which are shown in Table S3-1 [26–28].

$$EM_{Pr}^{food} = \sum_{i=1}^{N} \Delta Q_{loss,\,pr}^{i} \times C_{food}^{i} \quad \text{(S3-1)}$$

where EM_{Pr}^{food} is the amount of reduced GHG emissions in the production stage from food waste avoided by using this sensor-based DSL system (in Mt CO_2eq); $\Delta Q_{loss,pr}^{i}$ stands for the quantities of avoided food waste (in Mt); C_{food}^{i} refers to cradle-to-gate emission factors of food categories (in Mt CO_2eq/ton); and i, N represent the specific foodstuff and total food group, respectively.

Table S3-1: Cradle-to-gate emissions factors for different perishable foods

Food types	GHG emission factors of food production [kg CO_2eq/kg food] [26–28]
Vegetable	0.26
Fruit	0.893
Beef	60
Lamb	24
Pork	7
Chicken	6
Aquatic	5
Dairy	2.5
Eggs	4.01

3.2 Postharvest Handling and Packaging Stage

After harvest or slaughter, fresh food needed to be processed by either being stored or removing field heat. In this study, the sensor would be placed in a logistics box, thus enabling near-real-time data to be collected along the supply chain. Food products often required packaging that was expected to have a prolonged shelf life before delivering them to the market; however, packaging

materials can negatively impact the environment. Packaging emissions are not related to refrigeration and are considered only at the beginning of food cold chains. Referring to the study by Heller et al. (2019) [29], we assume that the packaging emission factor C_{pack} is proportional to the food emission factor C_{food} by the food-to-packaging ratio (FTP). In other words, $C_{pack} = \frac{C_{food}}{FTP}$. *The FTP ratio represents the cradle-to-grave GHG emissions ratio of the food product to its packaging*. In this paper, we assume the FTP to be the same as the cradle-to-gate ratio and calculate the mean packaging emission factors accordingly. The original data for FTP are presented in Table S3-2 of this Supplementary Materials. As a result, the sensor-based DSL system may have the potential to reduce the use of packaging materials due to potential FLW avoided. The emissions per unit of packaging for each food category are shown in Table S3-2. The reduced emissions from packaging (EM_{Pk}^{food}) are calculated in Eq. (S3-2).

$$EM_{Pk}^{food} = \sum_{i=1}^{N}(\Delta Q_{loss,pack}^{i} + \Delta Q_{loss,st}^{i} + \Delta Q_{loss,tr}^{i} + \Delta Q_{loss,\ di}^{i} + \Delta Q_{loss,re}^{i}) \times C_{pack}^{i} \qquad \text{(S3-2)}$$

where $\Delta Q_{loss,pack}^{i}$, $\Delta Q_{loss,st}^{i}$, $Q_{loss,tr}^{i}$, $\Delta Q_{loss,\ di}^{i}$, and $\Delta Q_{loss,re}^{i}$ represent the quantities of avoided FLW (in Mt) during the packaging, cold storage, transportation, distribution and retail stage.

Table S3-2: Packaging emissions per food category

**Food types**	**GHG emission factors of food production [kg CO2eq/kg food]**	**Emission factors of packaging [11,29] [kg CO2eq/kg food]**	**Food to packaging (FTP) ratio [11,29]**
Vegetable	0.26	0.14	2
Fruit	0.893	0.22	1.8
Beef	60	0.20	50
Lamb	24	0.19	50
Pork	7	0.20	5
Chicken	6	0.20	50
Aquatic	5	0.20	18
Dairy	2.5	0.20	2
Eggs	4.01	0.20	2

3.3 Cold Storage Stage

A common approach to extending the shelf life of perishable foods is to store them in temperature-controlled environments. The GHG emissions associated with refrigerated storage comprise three components: food loss, energy consumption, and refrigerant leakage. Since each food product is

stored under refrigeration, the energy required for the pre-cooling process is included at this stage. Accordingly, the GHG emissions of refrigerated storage are allocated among food loss, energy consumption, and refrigerant leakage.

As expressed in Eq. (S3-3), the net effect (EM_{St}^{food}) of mitigating waste during the cold storage stage is obtained by multiplying the total avoided FLW ($Q_{loss,St}^{i}$) by the emission factor (C_{food}^{i}) specific to each food category.

$$EM_{St}^{food} = \sum_{i=1}^{N} \Delta Q_{loss,St}^{i} \times C_{food}^{i} \quad \text{(S3-3)}$$

Energy consumption per unit of cold storage differs considerably by temperature-controlled types, capacities, and quantities of food products in stock. The Cold Chain Logistics Forum (2012) provides data on daily electricity consumption for refrigerated warehouses of various capacities in China. [11] We assume a 10-hour compressor operating period and an average food load of 75% capacity, the daily consumption is first divided by 0.75 to account for partial capacity utilization, and then divided by 10 to yield the hourly electricity consumption per ton of product (E_{St}). Eq. (S3-4) uses Em_{ele} to estimate the GHG emissions attributable to electricity consumption in the cold storage warehouse ($EM_{ele,st}$), where Em_{ele} [g CO_2e kWh^{-1}] represents the weighted life-cycle emission factor of China's electricity mix (See Table S3-3). This factor is determined by China's electricity generation structure and the emission intensities of individual power sources. [30,31] Further details on daily electricity consumption in cold storage warehouses and on China's electricity sources are available in Table S3-4.

$$EM_{ele,st} = \sum_{i=1}^{N} \Delta Q_{loss,\,St}^{i} \times E_{St} \times t_{St}^{i} \times Em_{ele} \quad \text{(S3-4)}$$

Regarding refrigerant leakage, we assumed a 20-year equipment lifetime, with an annual operational leakage rate of 8%, an installation leakage rate of 1%, and a disposal leakage rate of 5%. [32,33] We first multiply the annual leakage rate by the initial refrigerant charge of a commercial refrigerated warehouse (2,000–10,000 kg), then divide by the annual product throughput, which is approximately six times the warehouse capacity. [33,34] Installation and disposal leakages are similarly allocated based on the lifetime product throughput. The refrigerant leakage per kilogram of food product per hour ($leak_{st}$) is the sum of these leakage components over the respective

storage period. We then calculate the direct GHG emissions ($EM_{leak,\,st}$) using Eq. (S3-5), where t_{st}^{i} is the storage time and GWP is the average for the commonly used refrigerants in China (R22, R404a, and R134a). [35,36] Further details on refrigerant leakage allocation are provided in Supplementary Tables S3-4 and S3-5.

$$EM_{leak,\,st} = \sum_{i=1}^{N} \Delta Q_{st}^{i} \times leak_{st} \times t_{st}^{i} \times GWP_{ref} \quad \text{(S3-5)}$$

where $leak_{st}$ is the refrigerant leakage rate during storage (g/kg-h) and GWP_{ref} means the global warming potential of the refrigerant used in this study.

Table S3-3: China's energy-mix share

**Power source**	**Energy mix per years**					**Emission factor （gCO_2/kWh）**	**Weighted factors for 2020**
	2017	2018	2019	2020	2021		
Coal	62.50%	60.40%	58.80%	57.70%	56.80%	1230.0	709.71
Hydropower	20.80%	20.50%	21.40%	22.40%	22.80%	10.0	2.24
Nuclear	1.80%	2.00%	2.40%	2.50%	3.10%	17.0	0.425
Wind power	4.40%	5.20%	6.00%	7.40%	8.10%	46.0	3.404
Photovoltaic	1.30%	1.70%	2.10%	2.70%	3.70%	76.0	2.052
Natural gas	7.50%	8.30%	8.50%	8.30%	7.40%	856.0	71.048
Others	1.70%	1.90%	0.80%	1.00%	0.10%	97.0	0.97

Data source: Data obtained from China's National Energy Administration report. Note that, based on the report Global Energy and CO_2 Statistical Digest 2020, published by the International Energy Agency (IEA, 2020)*, China's average CO_2 emission factor for electricity was 0.848 tCO_2/MWh in 2018, compared to 0.838 tCO_2/MWh in 2019.*

Table S3-4: Cold-storage emissions allocation data

**Food types**	**GHG emission factors of food production [kg CO2eq/kg food]**	**Cold storage duration (h)**	**Unit power consumption (kWh/ton. h)**	**$kgCO_2eq$/ kWh**	**GWP**
Vegetable	0.26	24	0.0279	0.838	2351
Fruit	0.893	24	0.0279	0.838	2351
Beef	60	720	0.0279	0.838	2876
Lamb	24	720	0.0279	0.838	2876
Pork	7	720	0.0279	0.838	2876
Chicken	6	720	0.0279	0.838	2876
Aquatic	5	720	0.0279	0.838	2876
Dairy	2.5	24	0.0279	0.838	2351
Eggs	4.01	24	0.0279	0.838	2351

Table S3-5: Refrigerant-leakage allocation data

Cold chain Stage	Refrigerant	Average GWP	Annul leakage	Installation & disposal leakage	Charge capacity [kg]
Cold storage	R22/R134a/R404a (HT/MT)	2351	8%	6%	2,000 – 10,000
	R22/R404a (LT)	2876	8%	6%	2,000 – 10,000
Refrigerated transportation	R134a/R404a (HT/MT)	2621	8%	—	3 – 8
	R404a (LT)	3943	8%	—	3 – 8
Retail stores	R22/R134a/R404a (HT/MT)	2351	8%	—	2,000 – 10,000
	R22/R404a (LT)	2876	8%	—	2,000 – 10,000

Data source*: Data obtained from peer-reviewed literatures* [11,29,32,33,36,38]*. Note that HT, MT, LT denote high-temperature, medium-temperature, and low-temperature, respectively.*

3.4 Refrigerated Transportation Stage

The long-distance refrigerated transport of various food products took place from the origin of production sites to distribution centers. The source of GHG emissions consisted of three sub-processes: 1) food loss-related emissions, 2) energy-related emissions, and 3) refrigerant leakage-related emissions.

The reduction in food losses ($\Delta Q_{loss,tr}^{i}$) is determined by multiplying the transported volume of each perishable food category by its respective loss rate. The GHG emissions associated with these avoided losses during transit are then computed using Eq. (S3-6). Detailed loss rate data for each food category are provided in Tables S2–9 of the Supplementary Material. [24,25]

$$EM_{tr}^{food} = \sum_{i=1}^{N} \Delta Q_{loss,tr}^{i} \times C_{food}^{i} \quad \text{(S3–6)}$$

In this stage, we quantify the reduction in food losses (ΔQ_{tr}^{i}) by multiplying the transported volume of each perishable food category by its respective loss rate. During transit, the energy-related GHG emissions of refrigerated transportation are allocated on a per ton per kilometer basis, using the carbon emission factor (C_{tr}) for refrigerated vehicles in China (2.63 kg CO_2/L) [39], while also accounting for the average idling rate and the actual loading capacity of the vehicles. In the long-haul refrigerated transportation scenario, all examined food categories are assigned the same parameters, including a 63.40% share of the long-haul chain, an idle rate of 0.4, and a full load factor of 0.8. This uniformity ensures a consistent basis for comparing and analyzing their

associated GHG emissions. Drawing on the efficiency values derived from a UK study by Tassou et al. [40] and applying them to the Chinese context, Eq. (S3–7) calculates the transportation-specific GHG emissions ($EM_{ele,tr}$) under specified temperature conditions:

$$EM_{ele,tr} = \sum_{i=1}^{N} \Delta Q_{tr}^{i} \times C_{tr} \times D_{tr}/(1 - VER)/LR \tag{S3–7}$$

Here, ΔQ_{tr}^{i} is the amount of food transported for category i, C_{tr} is the carbon emission factor for refrigerated vehicles (kg CO_2eq/kg), D_{tr} is the average transport distance (km), VER represents the vehicle's idling or efficiency rate, and LR is the loading rate. The detailed parameter values are provided in Tables S3–6 and S3–7.

The initial transportation stage involves long-haul refrigerated trucking from the production source to the distribution center. We assume an annual refrigerant leakage rate of 8%, which is first multiplied by the initial refrigerant charge (3–8 kg) to determine the total annual leakage. [41,42] This total is then allocated to each kilogram of transported food by dividing it by both the truck's annual travel distance (assumed to be 60,000 km in China) and its carrying capacity (3–11 tons), thereby deriving the refrigerant leakage per kilogram of food per kilometer ($leak_{tr}$). [33,40] The specific travel distance for the first transportation segment (D_{tr}) for each food category is based on data from Huang et al. [43] With these parameters, the refrigerant transport-related GHG emissions ($EM_{leak,tr}$) can be calculated using Eq. (S3–8), where ΔQ_{tr}^{i} is the amount of food entering the transportation stage and GWP_{ref} is the average global warming potential of commonly used refrigerants (R134a/R404a). Further details are provided in Table S3–5.

$$EM_{leak,tr} = \sum_{i=1}^{N} \Delta Q_{tr}^{i} \times leak_{tr} \times D_{tr} \times GWP_{ref} \tag{S3–8}$$

Table S3-6: Refrigerated-transport emissions allocation data

Food types	GHG emission factors of food production [kg CO2eq/kg food]	Trans. distance (km) [43]	Emissions per ton-kilometre （$kgCO_2eq$/ton-km) [40,44]	Refrigerant leakage rate （kg/ton-km) [41,42]	GWP_{ref}
Veg.	0.26	950	0.145	1.30E-06	2351
Fruit	0.893	1040	0.145	1.30E-06	2351
Beef	60	590	0.191	1.30E-06	2876
Lamb	24	590	0.191	1.30E-06	2876
Pork	7	590	0.191	1.30E-06	2876
Chicken	6	590	0.191	1.30E-06	2876

Aquatic	5	850	0.191	1.30E-06	2876
Dairy	2.5	590	0.191	1.30E-06	2351
Eggs	4.01	590	0.191	1.30E-06	2351

3.5 Distribution Centre Stage

In this stage, the source of energy consumption came from storage locations and short-haul refrigerated transport from distribution centers to local markets. The detailed data associated with the food distributed is shown in Tables S3–7 to S3–9.

At the distribution center stage, the calculation methods mirror those employed for refrigerated storage at the cold storage warehouse stage. Equations quantify the GHG emissions associated with refrigerant leakage, electricity consumption, and food losses. In these equations, t_{dc}^{i} distinguishes the distribution center stage from the cold storage warehouse stage.

$$EM_{dc}^{food} = \sum_{i=1}^{N} \Delta Q_{loss,\,dc}^{i} \times C_{food}^{i} \tag{S3–9}$$

$$EM_{ele,dc} = \sum_{i=1}^{N} \Delta Q_{loss,\,dc}^{i} \times E_{dc} \times t_{dc}^{i} \times Em_{ele} \tag{S3–10}$$

$$EM_{leak,\,dc} = \sum_{i=1}^{N} \Delta Q_{dc}^{i} \times leak_{dc} \times t_{dc}^{i} \times GWP_{ref} \tag{S3–11}$$

In the second stage, short-haul refrigerated transportation conveys products from distribution centers to local markets. [43] In the short-haul refrigerated distribution segment, all examined food categories are allocated the same share of the short-haul chain (36.60%), idle rate (0.5), and full load factor (0.65). This uniformity ensures consistent assumptions when evaluating the GHG emissions attributable to short-distance refrigerated distribution. Furthermore, we apply the same methodology described in subsection 3.4 to determine the GHG emissions associated with this segment, incorporating the delivery distance and the share of short-haul delivery time to differentiate it from the preceding long-haul transportation stage.

$$EM_{delivery}^{food} = \sum_{i=1}^{N} \Delta Q_{loss,delivery}^{i} \times C_{food}^{i} \tag{S3–12}$$

$$EM_{ele,delivery} = \sum_{i=1}^{N} \Delta Q_{delivery}^{i} \times C_{delivery} \times D_{delivery}/(1 - VER)/LR \tag{S3–13}$$

$$EM_{leak,delivery} = \sum_{i=1}^{N} \Delta Q_{delivery}^{i} \times leak_{delivery} \times D_{delivery} \times GWP_{ref} \quad \text{(S3–14)}$$

Table S3-7: Distribution-phase emissions allocation data

Food types	GHG emission factors of food production [kg CO2eq/kg food]	Delivery distance (km) [43]	Emissions per ton-kilometre （kgCO2eq/ton-km)	Refrigerant leakage rate （kg/ton-km)	GWP
Veg.	0.26	50	0.222	1.30E-06	2351
Fruit	0.893	50	0.222	1.30E-06	2351
Beef	60	50	0.291	1.30E-06	2876
Lamb	24	50	0.291	1.30E-06	2876
Pork	7	50	0.291	1.30E-06	2876
Chicken	6	50	0.291	1.30E-06	2876
Aquatic	5	50	0.291	1.30E-06	2876
Dairy	2.5	50	0.275	1.30E-06	2351
Eggs	4.01	50	0.275	1.30E-06	2351

Table S3-8: Temporary-storage emissions allocation data

Food types	Storage time (h)	Unit power consumption（kWh/ ton.h）	kg/kWh	GWP
Veg.	48.00	0.0279	0.838	2351
Fruit	48.00	0.0279	0.838	2351
Beef	240.00	0.0279	0.838	2876
Lamb	240.00	0.0279	0.838	2876
Pork	240.00	0.0279	0.838	2876
Chicken	240.00	0.0279	0.838	2876
Aquatic	240.00	0.0279	0.838	2876
Dairy	48.00	0.0279	0.838	2351
Eggs	48.00	0.0279	0.838	2351

3.6 Retail Data

At this stage, GHG emissions were mitigated by reducing food losses, optimizing energy consumption, and minimizing refrigerant leakage. Detailed data supporting these findings are provided in Table S3-10.

Eq. (S3-15) computes retail food loss emissions (EM_{re}^{food}) by multiplying the quantity of food loss at the retail stage ($Q_{loss,re}^{i}$) by the corresponding loss emission factor (C_{food}^{i}).

$$EM_{re}^{food} = \sum_{i=1}^{N} \Delta Q_{loss,re}^{i} \times C_{food}^{i} \quad \text{(S3-15)}$$

At the retail stage, electricity consumption is partitioned into refrigeration-specific energy use (($E_{re,refri}$) and all other forms of energy consumption ($E_{re,\,other}$). [29] To estimate $E_{re,refri}$, we assume that low-temperature products are displayed in closed cases, while HT/MT products are stored in open cases. According to Fricke and Becker (2011) [45], open and closed display cases consume approximately 56 kWh/day/m³ and 40 kWh/day/m³, respectively. After adjusting these consumption values by considering the food bulk density, an average of about 24 kWh/ton-hour is derived. [46] Display case parameters and food bulk densities are detailed in Tables S3-11 and S3-12.

$$EM_{ele,re} = \sum_{i=1}^{N} \Delta Q_{loss,\,re}^{i} \times (E_{re,refri} + E_{re,\,other}) \times t_{re}^{i} \times Em_{ele} \quad \text{(S3-16)}$$

Referring to Dong et al. (2021), [11] we employ economic data on the Chinese retail sector (provided in Tables S3-13 to allocate $E_{re,\,other}$. Specifically, we calculate the ratio of the annual sales revenues for vegetables, fruits, meats, aquatic and dairy products in 2018 to the total sales revenue of the retail sector according to National Bureau of Statistics of China. [47] This ratio is then multiplied by the total energy consumption of the retail sector in 2018 and divided by the consumption volume of each food category to determine $E_{re,\,other}$. Finally, the GHG emissions associated with this portion of energy consumption ($EM_{ele,re}$) are computed using Eq. (S3-10), where $Q_{loss,re}^{i}$ is the volume of food products entering the retail stage, and t_{re}^{i} is the storage time at that stage.

Assuming that refrigerant leakage at the retail stage ($leak_{re}$) is equivalent to that observed in the cold storage warehouse ($leak_{st}$), we calculate $EM_{leak,\,re}$using Eq. (S3-11). In this calculation, Q_{re}^{i} represents the volume of food entering the retail stage and t_{re}^{i} denotes the duration for which it remains there. Further details on refrigerant leakage allocation and underlying assumptions can be found in Tables S3-5.

$$EM_{leak,\,re} = \sum_{i=1}^{N} \Delta Q_{re}^{i} \times leak_{re} \times t_{re}^{i} \times GWP_{ref} \quad \text{(S3-17)}$$

Table S3-9: Retail-phase emissions allocation data

Food types	GHG emission factors of food production [kg CO2eq/kg food]	On shelf duration (h)	Unit power consumption (kWh/ton.h)	kg/kWh	Refrigerant leakage rate (kg/ton.h)	GWP
Veg.	0.26	40	1.1	0.838	1.42123E-05	2351
Fruit	0.893	40	1.1	0.838	1.42123E-05	2351
Beef	60	240	0.41	0.838	1.42123E-05	2876
Lamb	24	240	0.41	0.838	1.42123E-05	2876
Pork	7	240	0.41	0.838	1.42123E-05	2876
Chicken	6	240	0.41	0.838	1.42123E-05	2876
Aquatic	5	240	0.53	0.838	1.42123E-05	2876
Dairy	2.5	48	1.1	0.838	1.42123E-05	2351
Eggs	4.01	48	1.1	0.838	1.42123E-05	2351

Table S3-10: Retail display-case energy consumption

Display cases [45]	Dimensions [m^3]	Energy Consumption [kWh/case-day]
Open case	6.35	56.5
Closed case	7.13	40.9

Note that products load 75% of total case capacity was considered.

Table S3-11: Bulk densities of food products

Product Category	Product Sub-Category	Density [kg/m^3] [46]	Density Used in Modelling [kg/m^3]
Vegetable	Carrot	600	460
	Chili	500	
	Green beans	530	
	Corn	650	
	Onion	550	
	Spinach leaves	80	
	lettuce	368	
Fruit	Apples	240	460
	Loquat	600	
	Pawpaw	560	
Meat	Pork	800	770
	Lamb	700	
	beef	820	
Aquatic	fish	600	600

Note that typical parameter values were derived from published references. The density selected for modeling approximates the mean reported values while also reflecting consumption preferences specific to the Chinese market.

Table S3-12: Non-refrigerated energy allocation

Product Category	Sales Revenue in 2018 [billion CNY]	Total Consumption in 2018 [ton]	Non-Refrigerated Energy Consumption [kWh/ton-h]
Vegetable	360 in total	72,699,298	0.0136
Fruit		134,096,018	0.0136
Meat	1477	53,722,130	0.107
Aquatic	284.19	15,907,332	0.070

Note that we consider 10 working hours every day to allocate to kWh/ton-h. Total revenue of retail sector in China in 2018: 33800 billion CNY. Total energy consumption of retail sector in China in 2018: 4.82 × 10^{11} kWh. [11]

3.7 Waste Treatment Stage

In this study, landfill was selected as the primary treatment method for near-expired food waste, reflecting its prevalence in China. The total GHG emissions associated with this process are quantified using Eq. (S3-18):

$$EM_{waste}^{food} = \sum_{i=1}^{N} \Delta Q_{loss,re}^{i} \times C_{waste}^{i} \tag{S3-18}$$

The inventory data for landfill processes, including pre-treatment, landfill gas (LFG) emissions, and leachate management, are presented in Table S3-14. Pre-treatment involves diesel and electricity consumption for food waste distribution, compaction, and administrative operations. LFG emissions are estimated based on biogenic carbon content, with raw gas fractions comprising 47% methane (CH_4) and 37% carbon dioxide (CO_2). A capture efficiency of 53% is applied, with 14.4% of the captured LFG utilized for electricity recovery at a conversion efficiency of 27.8%. Leachate production accounts for precipitation infiltration and landfill-specific characteristics, with treatment energy demands calculated at 0.216 kWh/m³. Oxidation of residual methane through managed landfill covers is modeled using a factor of 0.1, based on IPCC 2006 guidelines.

By integrating these parameters, the system reflects real-world landfill operations, providing a robust and transparent estimation of GHG emissions associated with food waste disposal. This approach highlights the environmental trade-offs of landfilling and supports the development of more sustainable food waste management strategies.

Table S3-13: Landfill-calculation inputs for 1 t food waste

Process	Parameter	Value	Unit	Reference/Comment

**Pre-Landfill Treatment**	Diesel demand in landfilling	1.3	L/ton FW	Utilized for distributing and compacting food waste.
	Electricity demand	0.015	kWh/ton FW	Required for administrative facilities.
	Heating oil demand	0.0045	L/ton FW	Used in administrative facilities.
**Lnadfill Gas (LFG) Engine**	Electricity demand for flaring and flue gas cleaning	1.6	kWh/ton FW	Based on the IWM-2 model.
	Electricity demand for LFG pumping	0.001365	kWh/kg waste	Comprises 0.00135 kWh/kg for gas pumps and 0.000015 kWh/kg for administrative facilities.
	Ratio of biogenic carbon in LFG	0.971	-	Ecoinvent - landfill report/
	Ratio of biogenic nitrogen in LFG	0.0644	-	Ecoinvent - landfill report.
	CH_4 volume fraction in raw LFG	47	%	Ecoinvent - landfill report.
	CO_2 volume fraction in raw LFG	37	%	Ecoinvent - landfill report.
	N_2 volume fraction in raw LFG	7.62	%	Ecoinvent - landfill report.
	CH_4 production in raw LFG generation	Calculated	m³	Formula: (Carbon mass in FW (g) × TS (%) × Carbon (%) × 0.971) ÷ 12 (g/mol) × 0.47 × 22.4 (L/mol) ÷ 1000
	CO_2 production in raw LFG generation	Calculated	m³	Formula: (Carbon mass in FW (g) × TS (%) × Carbon (%) × 0.971) ÷ 12 (g/mol) × 0.37 × 22.4 (L/mol) ÷ 1000.

	N_2 production in raw LFG generation	Calculated	m³	Nitrogen mass in FW (g) × TS (%) × Nitrogen (%) × 0.0644) ÷ 14 (g/mol) × 0.0762 × 22.4 (L/mol) ÷ 1000.
	Capture efficiency for LFG	53	%	Ecoinvent - landfill report.
	Ratio of LFG used for electricity recovery	14.4	%	Ecoinvent - landfill report.
	Electricity recovery efficiency	27.8	%	Ecoinvent - landfill report.
	Oxidation factor of CH_4 by managed cover with CH_4 oxidizing material	0.1	-	According to the 2006 IPCC Guidelines.
**Leachate Treatment**	Leachate volume	0.0025	m³/kg waste	Parameters: Rain infiltration rate (~500 mm/m²·a), average precipitation (~1000 mm/m²·a), actual infiltration water proportion (50%), landfill height (20 m), waste density (1000 kg/m³).
	Electricity consumption of the leachate treatment plant	0.216	kWh/m³	Ecoinvent - landfill report.

4 Life Cycle Inventory Analysis of Food Sensors

4.1 Goal and Scope

Goal: To quantify the life cycle GHG emissions associated with the deployment of IoT sensor systems for monitoring perishable foods throughout the post-farming supply chain up to retail stages in mainland China, with projections for future years.

Functionality: Food sensors are emerging as pivotal tools in the quest for enhanced food safety and quality assurance. These advanced devices detect specific analytes through sophisticated chemical or biological interactions, offering real-time insights into food integrity. The architecture of these sensors typically comprises four integrated components: target analyte, recognition element, signal transducer, and processor. Target analytes serve as molecular sentinels, signaling potential compromises in food safety or quality. Of particular concern are biotic contaminants (e.g., allergens, toxins, and pathogens) and abiotic contaminants (e.g., heavy metals and pesticides), which pose substantial risks to public health and food security globally. Recognition elements, whether derived from natural sources or engineered synthetically, are meticulously designed to bind with high specificity to target analytes. The selection of these elements is governed by stringent criteria, including exceptional specificity, sensitivity, stability, and biocompatibility with food matrices. Signal transducers convert molecular recognition events into quantifiable outputs, predominantly optical or electrical signals. These data undergo sophisticated processing algorithms and are presented in user-friendly formats, facilitating rapid decision-making in food production and distribution chains.

Functional Unit: One IoT sensor module monitoring temperature changes in a 100-liter intelligent container of perishable food.

System Boundary: See Fig. S4-1. It encompasses sensor production, operation, and end-of-life management, while excluding pre-farming stages and food production processes.

Geographical Boundary: Mainland China.

Temporal Boundary: The temporal boundary is set at 2020 as the base year, with projections for

2025 and 2030 based on estimated food production and consumption trends.

Key Assumptions: It includes temperature management for all perishable food, a 1:1 ratio between sensor modules and intelligent containers, and a standard 100-liter container volume for perishable food.

Data Requirements:

- Sensor specifications and material composition (as per Tables S4-1 to S4-3),
- Energy consumption during operation (as per Tables S4-4),
- E-waste treatment data (as per Tables S4-5),
- Number of sensors needed (as per Table S4-6), and
- Detailed container designs (as per Table S4-7).

Impact Categories: The impact categories assessed are limited to global warming potential (GWP) and energy use.

Allocation Procedures: It involves analyzing the ratio of sensor numbers required for varying masses of perishable food, accounting for different food types and their specific monitoring needs.

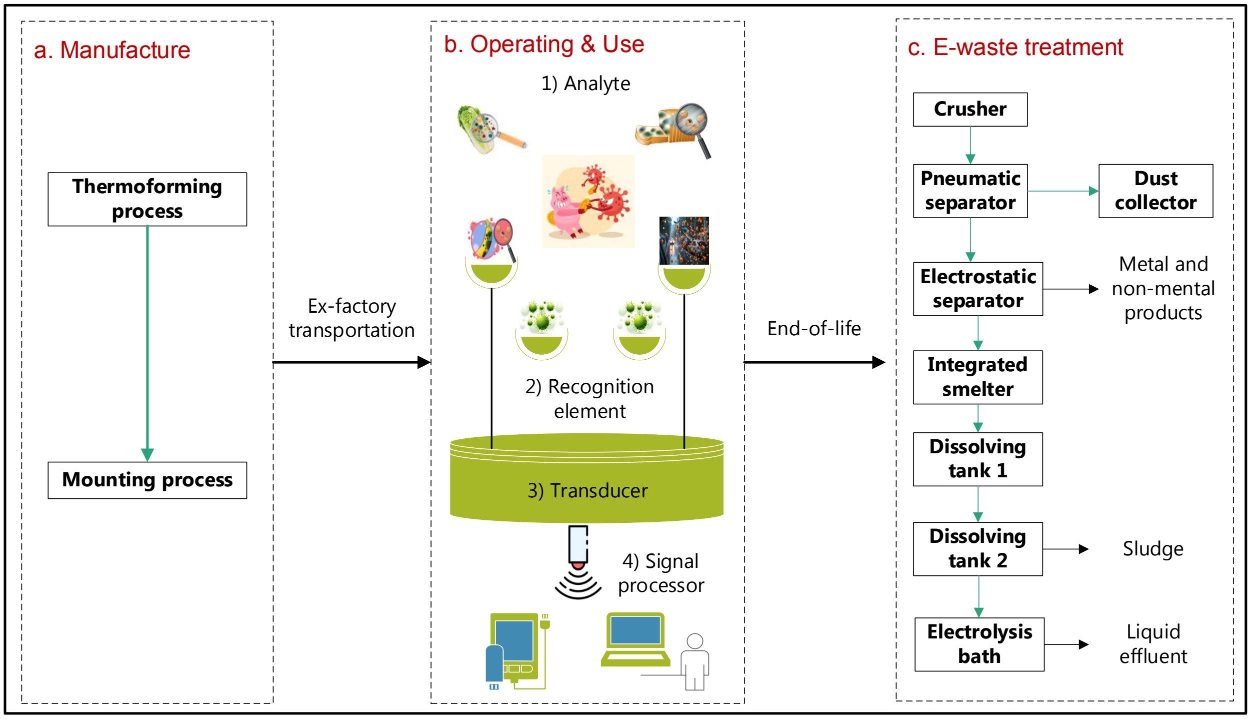

Figure S4-1: Food-sensor system boundaries **a)** The input flows of raw materials used in humidity-temperature sensor modules typically include noble metals, metals, metal oxides, resins, silicon, and ceramics. These materials are essential for the module's construction and functionality. **b)** A typical food sensor consists of four key components: analyte, recognition element, transducer, and signal processor, each playing a crucial role in detecting and processing the target information. The energy consumption of the sensor module varies depending on its operational state, being higher when activated for monitoring and significantly lower during sleep mode. **c)** The e-waste treatment process uses sulfuric, nitric, and hydrochloric acids as input materials. Emissions include particulate matter (PM_{10} and $PM_{2.5}$) captured by dust collectors, as well as hydrogen (H_2) and nitrogen oxides (NO/NO_2) released from dissolving tanks 1 and 2, respectively. Metal-bearing wastes separated by pneumatic or electrostatic methods contain Cr, Cd, Pd, Fe, and Al. Surplus wastes from the smelter include Pb and Zn, while the effluent from electrolysis baths in industrial treatment plants yields Au, Ag, Pd, Cu, and other valuable metals.

4.2 Inventory Analysis and Data Collection

The life cycle inventory (LCI) analysis of the sensor comprehensively evaluates the environmental impacts associated with its main components and functionalities. The sensor consists of a printed circuit board (PCB), central processing unit (CPU), real-time clock (RTC), humidity and temperature sensor, General Packet Radio Service (GPRS) module, Global Positioning System (GPS) module, gas sensors for carbon dioxide (CO_2) and hydrogen sulfide (H_2S) detection, and a lithium-ion battery. Detailed inventory data for each sensor module, including material inputs, energy consumption, and emissions, are provided in Tables S4-1 and S4-5. These data underpin the environmental performance assessment of the sensor, covering its production and operational phases within the food supply chain.

Table S4-1: Sensor module components (fruits, vegetables, dairy) [6]

Component category	Type	Weight (g)
Humidity-temperature sensor	SHT21	0.03
CO_2 sensor	COZIR-AH-1	0.036
Acceleration sensor	MMA8453QR1	0.03
Analog-to-digital converter	ADC08831M	0.08
Real time clock	DS2417P+	0.05
CPU	SEGGER 8.06.02 J-LINK 9-PIN CORTEX-M	6.52
GPS module	NEO-6M-0-001	1.03
GPRS module	USR-GM3	1.30
Lithium battery	703450	22.63
Printed circuit board	/	40.38

Table S4-2: Sensor module components (meat, fish, eggs)

Component category	Type	Weight (g)
Humidity-temperature sensor	SHT21	0.03
H_2S sensor	4HCiTiceL	5.00

Acceleration sensor	MMA8453QR1	0.03
Analog-to-digital converter	ADC08831M	0.08
Real time clock	DS2417P+	0.05
CPU	SEGGER 8.06.02 J-LINK 9-PIN CORTEX-M	6.52
GPS module	NEO-6M-0-001	1.03
GPRS module	USR-GM3	1.3
Lithium battery	703450	22.63
Printed circuit board	/	40.38

4.2.1 Manufacturing Stage

To model the GHG emissions from sensor manufacturing, the LCI analysis incorporates a detailed assessment of the raw materials involved in constructing the sensor. The selected humidity-temperature sensor is composed of a diverse array of materials, including inorganic substances such as silica and doped silicon, thermoset materials like epoxy resins, metals such as copper, and precious metals including gold and silver, alongside other organic compounds. The composition and quantities of these materials are outlined in Table S4-3, serving as the basis for estimating the energy and material flows associated with production.

The total GHG emissions from the manufacturing stage are calculated by multiplying the number of sensors produced (N_{sensor}) by the emissions factor per sensor (c_{sensor}), as described in Eq. S4-1. This emissions factor accounts for the environmental burdens associated with material extraction, thermoforming, and assembly processes. This approach ensures a systematic and transparent evaluation of the environmental impacts, providing a robust foundation for assessing the sustainability of sensor production within the life cycle framework.

$$EM_{Mf}^{senor} = N_{sensor} \times c_{sensor} \tag{S4-1}$$

Table S4-3: LCI of humidity-temperature sensor raw materials

Category	Substance	Content/%
Other inorganic materials	Silica	31.88
	Carbon black	0.11
	Doped Silicon	5.87
	Copper (II) oxide	0.05
Thermosets	Epoxy & Phenol Resin	4.69
	Epichlorohydrin-formaldehyde-phenol copolymer	0.05
Copper & its alloys	Copper	51.01
	Iron	1.23
	Zinc	0.06
	Phosphors	0.02

Precious metals	Silver	2.35
	Gold	0.87
	Palladium	0.01
Tin & its alloys	Tin	1.54
Other organic materials	Epoxy resin, epichlorohydrin-dimer fatty acid	0.05
	Butyrolactone, gamma-	0.05
	Poly(oxypropylene)diamine	0.05
	2,6-Diglycidyl phenyl allyl ether oligomer	0.05
	Organosilane	0.05
	Epoxy resin modifier	0.009

4.2.2 Operation Stage

During operation, IoT sensors alternate between working and sleeping states, consuming energy based on the duration spent in each mode. The average energy consumption per sensor module (E_{sensor}) is calculated as:

$$E_{sensor} = \left[(active\ power \times 0.8) + (sleep\ power \times 0.2)\right] \times \frac{24}{1000} \quad \text{(S4-2)}$$

For sensors consuming an average of 1.32 W in active mode and 0.14 W in sleep mode, E_{sensor} is determined using the proportion of time spent in each state. The total annual GHG emissions from sensor operation (EM_{Use}^{sensor}) are calculated using Eq. (S4-3):

$$EM_{Use}^{sensor} = \frac{N_{sensors} \times E_{sensor} \times GWP_{ele} \times 365}{2} \quad \text{(S4-3)}$$

here $N_{sensors}$ is the number of sensors deployed, and GWP_{ele} is the electricity emission factor (kg CO_2/kWh). This calculation reflects the energy consumed by sensors over a year.

To allocate GHG emissions across the supply chain stages (storage, transport, distribution, and retail), the proportion of time sensors operate in each stage is applied to EM_{Use}^{sensor}. The total operational time of sensors is linked to the average shelf life of monitored products and adjusted by their annual turnover cycles (See subsection 4.3).

The inventory data supporting these calculations, presented in Table S4-4, also includes additional system components such as GPS and GPRS modules, whose carbon footprints are scaled by a factor of 1.2 to ensure comprehensive coverage. Emissions from supercomputers supporting cloud-based traceability systems are evenly allocated to all sensors, adding 0.0073 kg CO_2-eq per module.

This methodology ensures accurate quantification of GHG emissions associated with sensor use, providing a transparent framework for evaluating the environmental impacts of IoT-enabled food supply chain monitoring systems.

Table S4-4: Sensor and component electricity consumption

**Sensor operation mode**	Sleeping Power/W	Measuring Power/W	**Comments**
Average	1.35E-01	1.32E+00	The energy consumed in one minute during a single data collection by a sensor with a power rating of *W, and its energy consumption per hour of continuous operation.
Min.	6.90E-02	5.83E-01	
Max.	2.00E-01	2.06E+00	
**Energy consumption**	Data collection (kWh)	Data transmission (kWh)	Note: To ensure a comprehensive assessment, a scaling factor of 1.2 was applied to account for additional components of the sensor system (e.g., GPS, GPRS and ADC) whose carbon footprints could not be precisely quantified. [5] Moreover, the emissions associated with the operation of supercomputers supporting the cloud-based and traceability systems were integrated into the overall carbon footprint of the sensor system. These emissions were allocated evenly across the sensor modules, contributing an additional 0.0073 kg CO_2-eq per module.
Average	9.26E-03	5.95E-05	
Min.	4.65E-03		
Max.	5.54E-03		
**Operation time**	The total operating time of the sensor is determined based on the average shelf life of various products (See table S4-6), with a total service life of 5 years. Assuming an annual operating period of 365 days, the specific usage frequency is calculated according to the annual operating cycles of the turnover boxes (see Section 4.3).		

4.2.3 Waste Treatment Stage

The default values in Table S4-5 provide an LCI analysis for quantifying the GHG emissions and resource recovery associated with the end-of-life management of integrated sensor modules. These values encompass energy inputs, material recovery efficiencies, and the use of auxiliary materials across key processes, such as crushing, smelting, leaching, incineration, and landfilling, ensuring a comprehensive evaluation of the waste treatment stage.

The GHG emissions during the waste treatment phase ($EM_{recycle}^{sensor}$) are calculated using Eq. (S4-7)

by multiplying the total number of sensors ($N_{sensors}$) by the standardized GWP value for food sensors during recycling ($C_{recycle}$), adjusted for the service life ($1/n$) of the sensors:

$$EM_{recycle}^{sensor} = N_{sensors} \times 1/n \times C_{recycle} \quad \text{(S4-7)}$$

Table S4-5: LCI of sensor-module treatment and recycling

Process	Description	Default value [48,49]
Crusher	Electricity (kWh)	160
	Loss rate of materials (%)	1
Pneumatic/ Electrostatic separator	Electricity (kWh)	56.7
	Recovery efficiencies for Fe and Al (%)	95
	Loss rate of materials (%)	0.5
Integrated smelter	Electricity (kWh)	197.2
	Recovery efficiencies for lead (%)	95
	Recovery efficiencies for zinc (%)	97
Leaching/ electrolysis	Electricity (kWh)	299.2
	Leaching efficiencies for gold (%)	97
	Leaching efficiencies for silver (%)	98
	Leaching efficiencies for palladium (%)	93
	Leaching efficiencies for copper (%)	100
	Leaching efficiencies for Ni (%)	99
	Electrolytic reduction efficiencies (%)	100
	Acid usage (liters/kg metal)	20
	Density of acid (kg/L)	1.8 (sulfuric acid), 1.4 (nitric acid), 1.2 (hydrochloric acid)
	Usage of H_2SO_4/L	321
	Usage of HNO_3/L	836
	Usage of HCl/L	2508
Incineration	Activated carbon (g/kg waste)	1.2
	$Ca(OH)_2$ (g/kg waste)	4
	Natural gas (m^3/kg waste)	0.042
	Electricity (kWh/ kg waste)	0.133
	Water addition (kg/kg waste)	0.09
	Incineration ash generation (kg/kg waste)	0.08
Landfill	Electricity (kWh/kg waste)	3.59×10^{-4}
	Diesel (mL/kg waste)	0.32
	Water addition (g/kg waste)	94
	LDPE (m^3/kg waste)	6.46×10^{-8}
	Sand (m^3/ kg waste)	3.51×10^{-4}
	Pesticide (mg/kg waste)	32.3
Wastewater treatment	Electricity (kwh/t wastewater)	0.375
	Activated carbon (kg/t wastewater)	0.1088
	Aluminum sulfate (kg/t wastewater)	15

4.3 Estimation of Sensors Used for Perishable Food Systems

To calculate the national GHG emissions produced by sensors monitoring perishable foods, we first estimate the number of sensors required per year (N_{year}^{sensor}) using Eq. (S4-8):

$$N_{year}^{sensor} = \sum_{i=1}^{6} N_{\frac{sensor}{d}}^{i} \bullet \frac{SL^{i}}{2} \quad \text{(S4-8)}$$

where $N_{sensor/d}^{i}$ is calculated using Eq. (S4-9):

$$N_{\frac{sensor}{d}}^{i} = \frac{W_{d}^{i}}{C^{i}} = W_{year}^{i} \times \frac{1000}{365.\, C^{i}} \quad \text{(S4-9)}$$

Using these equations, the required number of sensors for each perishable food category is determined by the following steps:

1. Calculate the daily production weight ($W_{d}^{i} = W_{year}^{i} / \, 36^{5}$) for each food category.
2. Divide W_{d}^{i} by the weight capacity per intelligent container (C^{i}), derived from the food density and container volume, to estimate $N_{sensor/d}^{i}$.
3. Multiply $N_{sensor/d}^{i}$ by half the average shelf life ($\frac{SL^{i}}{2}$) to account for continuous food consumption.

For example, Table S4-6 shows that for vegetables, with an annual demand of 79.47 million tons, a shelf life of 46 days, and a container weight capacity of 6.9 kg, the annual sensor requirement ($\times 10^{5}$ units) is:

$$N_{sensor/d}^{vegetable} = \frac{79{,}471 \cdot 1000}{365 \cdot 6.9} \approx 31{,}532$$

$$N_{year}^{vegetable} = 31{,}532 \cdot \frac{46}{2} \approx 217{,}730$$

Summing across all food categories, the total annual sensor requirement is approximately 798.57×10^{5} units. The calculation incorporates assumptions that each intelligent container requires one sensor (Table S4-7) and that all perishable foodstuffs are monitored for temperature. Food production data is sourced from the *China Agriculture Outlook Report (2018–2027)*, [50] while shelf life and container parameters are based on authoritative guidelines (*Food Cold Chain Logistics -*

Temperature Control Requirements for Storage, Transport and Distribution). [17] This systematic approach ensures transparency and consistency in estimating the number of sensors required for national-scale monitoring.

Table S4-6: Estimated sensor counts per food type

	Food demand/ ×10^5 tons	Shelf life/ day	Quantities of sensors/ ×10^5 units
**Vegetable**	79,4717	46	217,73
**Fruit**	66,984	44	115,35
**Beef**	4,0992	180	15,55
**Lamb**	3,0012	180	11,38
**Pork**	25,0893	180	95,18
**Chicken**	14,4021	180	54,63
**Aquatics**	40,579	180	212,89
**Dairy**	23,7582	18	11,72
**Eggs**	27,0504	45	64,13
**Total**			798,57

Notes: *The data on food production is from the China Agriculture Outlook Report (2018-2027).* [50] *Representative data on the shelf life of these food categories were sourced from 'Food Cold Chain Logistics - Temperature Control Requirements for Storage, Transport and Distribution'.* [17]

Table S4-7: Intelligent container design specifications

Parameter	Value
Volume (L)	30
Length × width × height (m)	0.6×0.4×0.125
Pallet load/numbers of boxes	2×2×11=44
Space utilization of intelligent container (%)	33.3
Sensor quantity in container	1
Location of sensor	Top level in the box
Assembly method	Electric soldering iron
Life span/months	36

Table S4-8: Food density (weight per unit volume)

Food type	Food density; kg/m^3	Food density calculated; kg/30L
Vegetables	230	6.9
Fruit	350	10.5
Meat	650	19.5
Aquatic	470	14.1
Dairy	500	15
Eggs	260	7.8

5 Development of Scenario Settings

China's food production and diet structure

China's food production is projected to follow an upward trend from 2020 to 2025, driven by advancements in agricultural technologies and demographic factors, before transitioning to a gradual decline between 2025 and 2030 due to a slowing population growth rate and evolving dietary preferences. Significant shifts in diet structure are expected to influence production patterns. For example, pork production is anticipated to increase by 40.3% in 2025 compared to 2020, followed by a moderated growth rate of 3.9% by 2030 compared to 2025. Similarly, beef and lamb production are expected to experience steady growth over the next decade. In contrast, dairy production is projected to grow rapidly, increasing by 12.5% in 2025 relative to 2020 and a further 10% in 2030 compared to 2025. Conversely, the production of fruits, vegetables, and aquatic products is expected to see slower growth rates, reflecting changing dietary patterns and market dynamics.

These projections are derived from the China Agriculture Outlook Report (2018–2027), [50] which provides authoritative insights into national food production trends and shifts in dietary preferences. This report offers a reliable basis for estimating future changes in production and consumption patterns.

The circulation rate of cold chains is linked to the demand for fresh food

The circulation rate of cold chains is projected to improve significantly due to increasing demand for fresh food and government-led infrastructure upgrades. Currently, China's cold chain logistics system lags behind developed countries, limiting the widespread adoption of IoT sensor-based DSL systems to mitigate food loss and waste (FLW). However, ongoing initiatives to modernize the infrastructure are expected to drive substantial improvements. By 2025, the circulation rates for fruit and vegetables, meat, and aquatic products are projected to reach 30%, 85%, and 85%, respectively, compared to 2020 levels. By 2030, these rates are expected to further increase to 50% for fruit and vegetables and 90% for meat and aquatic products.

These estimates are based on the Development Plan of Cold Chain Logistics for the "14th Five-Year Plan", [51] which outlines the strategic priorities for upgrading China's cold chain logistics systems to enhance the quality and safety of perishable food products.

Energy demand and food refrigeration

A critical factor in food refrigeration is the carbon intensity of the electricity grid, which significantly impacts the climate change mitigation potential of IoT sensor-based DSL systems. Projections suggest that by 2030, the share of non-fossil energy in China's energy mix will increase to 27%, while the share of coal will decrease to below 50%, reflecting a shift toward cleaner energy sources. This transition is expected to reduce the carbon emission intensity of electricity used for food refrigeration by 20% by 2025 and 50% by 2030 compared to 2020 levels.

These projections are derived from the Sino-German Energy Partnership, which provides comprehensive data on China's clean energy transition. The findings were incorporated into this study to model the carbon intensity of food refrigeration systems in the future.

Table S5-1: 2020 food production, circulation, demand (China)

Food Category	2020		
	Production (10×10^3 tons)	Circulation rate of food cold chains (%)	Demand of food cold chains (10×10^3 tons)
Vegetable	72,247	11	7,780
Fruit	27,910	24	6,696
Beef	672	61	410
Lamb	492	61	300
Pork	4,113	61	2,509
Chicken	2,361	61	1,440
Seafood	6,545	62	4,055
Dairy	3,546	67	2,392
Egg	3,468	78	2,710
Total	121,354		28,293

Notes: *The data of circulation of the FCC is from the Development Plan of Cold Chain Logistics for the "14th Five-Year Plan"* [51]*. The data on food production is from the China Agriculture Outlook Report (2018-2027)* [50].

Table S5-2: 2025 food production, circulation, demand (China)

	2025		
Food Category	Production (10×10^3 tons)	Circulation rate of the FCC (%)	Demand of the FCC (10×10^3 tons)
Vegetable	76,740	30	23,022
Fruit	31,523	30	9,457
Beef	729	85	620
Lamb	533	85	453
Pork	5,772	85	4,906
Chicken	2,527	85	2,148
Seafood	6,787	85	5,769

**Dairy**	3,989	85	3,391
**Egg**	3,551	85	3,018
**Total**	132,151		52,783.7

Notes: *The data of circulation of the FCC is from the Development Plan of Cold Chain Logistics for the "14th Five-Year Plan"* [51]. *The data on food production is from the China Agriculture Outlook Report (2018-2027)* [50].

Table S5-3: 2030 food production, circulation, demand (China)

**2030**			
**Food Category**	Production (10×10^3 tons)	Circulation rate of the FCC (%)	Demand of the FCC (10×10^3 tons)
**Vegetable**	79,795	50	39,898
**Fruit**	34,685	50	17,343
**Beef**	790	90	711
**Lamb**	576	90	518
**Pork**	5,998	90	5,398
**Chicken**	2,555	90	2,300
**Seafood**	6,991	90	6,292
**Dairy**	4,389	90	3,950
**Egg**	3,650	90	3,285
**Total**	139,429		79,694

Notes: *The data of circulation of the FCC is from the Development Plan of Cold Chain Logistics for the "14th Five-Year Plan"* [51]. *The data on food production is from the China Agriculture Outlook Report (2018-2027)* [50].

References

(1) Clark, M.; Springmann, M.; Rayner, M.; Scarborough, P.; Hill, J.; Tilman, D.; Macdiarmid, J. I.; Fanzo, J.; Bandy, L.; Harrington, R. A. Estimating the Environmental Impacts of 57,000 Food Products. Proc. Natl. Acad. Sci. U.S.A. **2022***, 119* (50), e2205763119. https://doi.org/10.1073/pnas.

(2) Crippa, M.; Solazzo, E.; Guizzardi, D.; Monforti-Ferrario, F.; Tubiello, F. N.; Leip, A. Food Systems Are Responsible for a Third of Global Anthropogenic GHG Emissions. *Nat Food* **2021**, *2* (3), 198–209. https://doi.org/10.1038/s43016-021-00225-9.

(3) FAO. *Food Wastage Footprint Impacts on Natural Resources Summary Report*; Food and Agricultural Organization of the United Nations: Rome, **2013**. Available online: https://www.fao.org/3/i3347e/i3347e.pdf (accessed 2024-06-24)

(4) Amicarelli, V.; Bux, C. Food Waste Measurement toward a Fair, Healthy and Environmental-Friendly Food System: A Critical Review. *Br. Food J.* **2020**, *123* (8), 2907–2935. https://doi.org/10.1108/BFJ-07-2020-0658.

(5) Luo, Z.; Zhu, J.; Sun, T.; Liu, Y.; Ren, S.; Tong, H.; Yu, L.; Fei, X.; Yin, K. Application of the IoT in the Food Supply Chain─From the Perspective of Carbon Mitigation. *Environ Sci Technol* **2022**, *56* (15), 10567–10576. https://doi.org/10.1021/acs.est.2c02117.

(6) Zhu, J.; Luo, Z.; Liu, Y.; Tong, H.; Yin, K. Environmental Perspectives for Food Loss Reduction via Smart Sensors: A Global Life Cycle Assessment. *J Clean Prod* **2022**, *374*, 133852. https://doi.org/10.1016/j.jclepro.2022.133852.

(7) Holden, N. M.; White, E. P.; Lange, Matthew. C.; Oldfield, T. L. Review of the Sustainability of Food Systems and Transition Using the Internet of Food. *NPJ Sci Food* **2018**, *2* (1), 7. https://doi.org/10.1038/s41538-018-0027-3.

(8) Salinas Segura, A.; Thiesse, F. A Comparison of Sensor-Based Issuing Policies in the Perishables Supply Chain. *Int. J. RF Technol. Res. Appl.* **2017**, *8* (3), 123–141. https://doi.org/10.3233/RFT-171672.

(9) Labuza, T. P.; Taoukis, P. S. The Relationship between Processing and Shelf-Life. *Foods for the '90s.* Elsevier Applied Science: London 1990, pp 73–106.

(10) Singh, T. K.; Cadwallader, K. R. *Ways of Measuring Shelf-Life and Spoilage*; Woodhead Publishing Limited, 2004; pp 165–198. https://doi.org/10.1533/9781855739024.2.165.

(11) Dong, Y.; Miller, S. A. Assessing the Lifecycle Greenhouse Gas (GHG) Emissions of Perishable Food Products Delivered by the Cold Chain in China. *J Clean Prod* **2021**, *303*, 126982. https://doi.org/10.1016/j.jclepro.2021.126982.

(12) Ndraha, N.; Sung, W. C.; Hsiao, H. I. Evaluation of the Cold Chain Management Options to Preserve the Shelf Life of Frozen Shrimps: A Case Study in the Home Delivery Services in Taiwan. *J Food Eng* **2019**, *242*, 21–30. https://doi.org/10.1016/j.jfoodeng.2018.08.010.

(13) Spada, A.; Conte, A.; Del Nobile, M. A. The Influence of Shelf Life on Food Waste: A Model-Based Approach by Empirical Market Evidence. *J Clean Prod* **2018**, *172*, 3410–3414. https://doi.org/10.1016/j.jclepro.2017.11.071.

(14) Jedermann, R.; Geyer, M.; Praeger, U.; Lang, W. Sea Transport of Bananas in Containers - Parameter Identification for a Temperature Model. *J Food Eng* **2013**, *115* (3), 330–338. https://doi.org/10.1016/j.jfoodeng.2012.10.039.

(15) Yi, T.; Jing, X.; Hong, X. Prediction Model of Shelf Life of Trichiurus Haumela Using an Electric Nose. *Transactions of the CSAE* **2010**, *26* (2), 356–360. https://doi.org/doi：10.3969/j.issn.1002-6819.2010.02.062.

(16) Göransson, M.; Nilsson, F.; Jevinger. Temperature Performance and Food Shelf-Life Accuracy in Cold Food Supply Chains – Insights from Multiple Field Studies. *Food Control* **2018**, *86*, 332–341. https://doi.org/10.1016/j.foodcont.2017.10.029.

(17) DB35/T 1805—2018. *Food Cold Chain Logistics — Temperature Control Requirements for*

Storage, Transportation and Sale; Fujian Quality and Technical Supervision Bureau: Fujian, 2019.
(18) Lu, S.; Cheng, G.; Li, T.; Xue, L.; Liu, X.; Huang, J.; Liu, G. Quantifying Supply Chain Food Loss in China with Primary Data: A Large-Scale, Field-Survey Based Analysis for Staple Food, Vegetables, and Fruits. *Resour Conserv Recycl* **2022**, *177*, 106006. https://doi.org/10.1016/j.resconrec.2021.106006.
(19) Wang, R.; Liu, G.; Zhou, L.; Yang, Z.; Tang, Z.; Lu, S.; Zhao, M.; Sun, H.; Ma, C.; Cheng, G. Quantifying Food Loss along the Animal Products Supply Chain in China with Large-Scale Field-Survey Based Primary Data. *Resour Conserv Recycl* **2023**, *188*, 106685. https://doi.org/10.1016/j.resconrec.2022.106685.
(20) Likar, K.; Jevšnik, M. Cold Chain Maintaining in Food Trade. *Food Control* **2006**, *17* (2), 108–113. https://doi.org/10.1016/J.FOODCONT.2004.09.009.
(21) Derens, E.; Palagos, B.; Guilpart, J. The Cold Chain of Chilled Products under Supervision in France. In *Proceedings of 16th World Congress of Food Science and Technology*, Montpellier, France, **2006**; pp 51–64. https://doi.org/10.1051/iufost:20060823.
(22) Nunes, M. C. N.; Emond, J. P.; Rauth, M.; Dea, S.; Chau, K. V. Environmental Conditions Encountered during Typical Consumer Retail Display Affect Fruit and Vegetable Quality and Waste. *Postharvest Biol Technol* **2009**, *51* (2), 232–241. https://doi.org/10.1016/j.postharvbio.2008.07.016.
(23) Zou, Y.; Wu, J.; Liu, G.; Piron, M.; Fedele, A.; Antonio, S.; Manzardo, A. Examining the Trade-Offs in Potential Retail Benefits of Different Expiration Date Modes: Insights into Multidimensional Scenarios. *Resour. Conserv. Recycl.* **2022**, *186*, 106511. https://doi.org/10.1016/j.resconrec.2022.106511.
(24) Wang, R.; Liu, G.; Zhou, L.; Yang, Z.; Tang, Z.; Lu, S.; Zhao, M.; Sun, H.; Ma, C.; Cheng, G. Quantifying Food Loss along the Animal Products Supply Chain in China with Large-Scale Field-Survey Based Primary Data. *Resour Conserv Recycl* **2023**, *188*, 106685. https://doi.org/10.1016/j.resconrec.2022.106685.
(25) Lu, S.; Cheng, G.; Li, T.; Xue, L.; Liu, X.; Huang, J.; Liu, G. Quantifying Supply Chain Food Loss in China with Primary Data: A Large-Scale, Field-Survey Based Analysis for Staple Food, Vegetables, and Fruits. *Resour Conserv Recycl* **2022**, *177*, 106006. https://doi.org/10.1016/j.resconrec.2021.106006.
(26) Porter, S. D.; Reay, D. S.; Higgins, P.; Bomberg, E. A Half-Century of Production-Phase Greenhouse Gas Emissions from Food Loss & Waste in the Global Food Supply Chain. *Sci. Total Environ.* **2016**, *571*, 721–729. https://doi.org/10.1016/j.scitotenv.2016.07.041.
(27) Hu, G.; Mu, X.; Xu, M.; Miller, S. A. Potentials of GHG Emission Reductions from Cold Chain Systems: Case Studies of China and the United States. *J Clean Prod* **2019**, *239*, 118053. https://doi.org/10.1016/j.jclepro.2019.118053.
(28) Dong, Y.; Xu, M.; Miller, S. A. Overview of Cold Chain Development in China and Methods of Studying Its Environmental Impacts. *Environ Res Commun* **2021**, *2* (12), 122002. https://doi.org/10.1088/2515-7620/abd622.
(29) Heller, M. C.; Selke, S. E. M.; Keoleian, G. A. Mapping the Influence of Food Waste in Food Packaging Environmental Performance Assessments. *J Ind Ecol* **2019**, *23* (2), 480–495. https://doi.org/10.1111/jiec.12743.
(30) Li, X.; Chalvatzis, K. J.; Pappas, D. China's Electricity Emission Intensity in 2020 - An Analysis at Provincial Level. In *Energy Procedia*; Elsevier Ltd, 2017; Vol. 142, pp 2779–2785. https://doi.org/10.1016/j.egypro.2017.12.421.
(31) International Energy Agency (IEA). *Electricity generation by source in China [Online].* IEA: Paris, **2020**. Available online: https://www.iea.org/data-and-statistics/data-tools/electricity-information (accessed 2024-06-24).
(32) Hoang, H. M.; Brown, T.; Indergard, E.; Leducq, D.; Alvarez, G. Life Cycle Assessment of Salmon Cold Chains: Comparison between Chilling and Superchilling Technologies. *J Clean Prod* **2016**, *126*, 363–372. https://doi.org/10.1016/j.jclepro.2016.03.049.
(33) International Institute of Refrigeration; UN Environment. *Cold Chain Brief on Cold Storage and*

Refrigerated Warehouse; 3rd ed.; IIR: Paris, 2018. https://doi.org/https://www.unep.org/ozonaction/resources/publication/cold-chain-technology-brief-cold-storage-and-refrigerated-warehouse. (accessed 2024-06-24)
(34) China Federation of Logistics & Purchasing. *Harbor cold warehouse report (In Chinese) [Online]*. 2020. Available online: http://www.chinawuliu.com.cn/zx/20200625/I011.htm (accessed 2024-06-24)
(35) Zhao, H.; Liu, S.; Tian, C.; Yan, G.; Wang, D. An Overview of Current Status of Cold Chain in China. *Int. J. Refrig.* **2018**, *88*, 483–495. https://doi.org/10.1016/j.ijrefrig.2018.02.024.
(36) M. Zhang; M. Lu; Y. Zhou. An Overview of R22 Refrigerant Substitution in China. *Air & Waste Management Association (A&WMA), EM Magazine*. **2019**, *51* (9), A1–A4. https://www.awma.org/emsept19 (accessed 2024-12-04).
(37) IEA. *Global Energy Review 2020*; IEA: Paris, 2021. Available online: https://www.iea.org/reports/global-energy-review-2020 (accessed 2024-06-24)
(38) Heard, B. R.; Bandekar, M.; Vassar, B.; Miller, S. A. Comparison of Life Cycle Environmental Impacts from Meal Kits and Grocery Store Meals. *Resour Conserv Recycl* **2019**, *147*, 189–200. https://doi.org/10.1016/j.resconrec.2019.04.008.
(39) Li, H.; Li, D.; Jiang, D. Optimising the Configuration of Food Supply Chains. *Int J Prod Res* **2021**, *59* (12), 3722–3746. https://doi.org/10.1080/00207543.2020.1751337.
(40) Tassou, S. A.; De-Lille, G.; Ge, Y. T. Food Transport Refrigeration - Approaches to Reduce Energy Consumption and Environmental Impacts of Road Transport. *Appl Therm Eng* **2009**, *29* (8–9), 1467–1477. https://doi.org/10.1016/j.applthermaleng.2008.06.027.
(41) Wu, J.; Liu, G.; Marson, A.; Fedele, A.; Scipioni, A.; Manzardo, A. Mitigating Environmental Burden of the Refrigerated Transportation Sector: Carbon Footprint Comparisons of Commonly Used Refrigeration Systems and Alternative Cold Storage Systems. *J Clean Prod* **2022**, *372*, 133514. https://doi.org/10.1016/j.jclepro.2022.133514.
(42) Wu, J.; Li, Q.; Liu, G.; Xie, R.; Zou, Y.; Scipioni, A.; Manzardo, A. Evaluating the Impact of Refrigerated Transport Trucks in China on Climate Change from the Life Cycle Perspective. *Environ Impact Assess Rev* **2022**, *97*, 106866. https://doi.org/10.1016/j.eiar.2022.106866.
(43) Jingnan, H.; Danzhe, L.; Qin, X. Food Miles of Chinese Big Cities and Its Planning Implications: Taking Wuhan as an Example. *Urban Plan. Int.* **2014**, 29 (5), 101–106.
(44) Liu, G.; Hu, J.; Yang, Y.; Xia, S.; Lim, M. K. Vehicle Routing Problem in Cold Chain Logistics: A Joint Distribution Model with Carbon Trading Mechanisms. *Resour Conserv Recycl* **2020**, *156*, 104715. https://doi.org/10.1016/j.resconrec.2020.104715.
(45) B.A. Fricke; B.R. Becker. Comparison of Vertical Display Cases: Energy and productivity Impacts of Glass Doors versus Open Vertical Display Cases. In *Building Simulation: Proceedings of the 12th Conference*; 2011; Vol. 847e858, p 117.
(46) Charrondiere, U. R.; Haytowitz, D.; Stadlmayr, B. FAO/ INFOODS Databases: Density Database Version 2.0. *Database* **2012**, *24*, e00056.
(47) *National Bureau of Statistics of China China Statistical Yearbook 2020 [Online]. National Bureau of Statistics of China: Beijing, 2020. Available online: http://www.stats.gov.cn/tjsj/ndsj/2020/indexeh.htm (accessed 2024-06-24)*
(48) Xue, M.; Kendall, A.; Xu, Z.; Schoenung, J. M. Waste Management of Printed Wiring Boards: A Life Cycle Assessment of the Metals Recycling Chain from Liberation through Refining. *Environ Sci Technol* **2015**, *49* (2), 940–947. https://doi.org/10.1021/es504750q.
(49) Hong, J.; Shi, W.; Wang, Y.; Chen, W.; Li, X. Life Cycle Assessment of Electronic Waste Treatment. *Waste Manage.* **2015**, *38* (1), 357–365. https://doi.org/10.1016/j.wasman.2014.12.022.
(50) Yu, L.; Wang, Z.; Zhu, X.; Xu, S.; Liu, G.; Li, G.; Yang, J.; Wu, L.; Qin, F.; Zhang, X.; Dong, C.; Huang, H. *China Agricultural Outlook (2018-2027)*; Beijing, 2018. Available online: https://aocm.agri-outlook.cn https://aocm.agri-outlook.cn (accessed 2024-06-24).
(51) The State Council of the People's Republic of China. *Development Plan of Cold Chain Logistics for the "14th Five-Year Plan."* General Office of the State Council: Beijing, 2021. Available online:

https://www.gov.cn/zhengce/content/2021-12/12/content_5660244.htm (accessed 2024-06-24).
(52) Yin, Y.; Hove, A.; Deutsche Gesellschaft für; Internationale Zusammenarbeit (GIZ) GmbH. *China Energy Transition Status Report 2020*; Beijing, 2021.
